# [3 + 2] Cycloaddition with photogenerated azomethine ylides in β-cyclodextrin

**DOI:** 10.3762/bjoc.16.110

**Published:** 2020-06-12

**Authors:** Margareta Sohora, Leo Mandić, Nikola Basarić

**Affiliations:** 1 Department of Organic Chemistry and Biochemistry, Ruđer Bošković Institute, Bijenička cesta 54, 10 000 Zagreb, Croatia. Fax: +385 1 4680 195; Tel: +385 12 4561 141; 2 Department of Material Chemistry, Ruđer Bošković Institute, Bijenička cesta 54, 10 000 Zagreb, Croatia

**Keywords:** [3 + 2] cycloadditions, β-cyclodextrin, inclusion complexes, photochemistry, phthalimides

## Abstract

Stability constants for the inclusion complexes of cyclohexylphthalimide **2** and adamantylphthalimide **3** with β-cyclodextrin (β-CD) were determined by ^1^H NMR titration, *K* = 190 ± 50 M^−1^, and *K* = 2600 ± 600 M^−1^, respectively. Photochemical reactivity of the inclusion complexes **2@β-CD** and **3@β-CD** was investigated, and we found out that β-CD does not affect the decarboxylation efficiency, while it affects the subsequent photochemical H-abstraction, resulting in different product distribution upon irradiation in the presence of β-CD. The formation of ternary complexes with acrylonitrile (AN) and **2@β-CD** or **3@β-CD** was also essayed by ^1^H NMR. Although the formation of such complexes was suggested, stability constants could not be determined. Irradiation of **2@β-CD** in the presence of AN in aqueous solution where cycloadduct **7** was formed highly suggests that decarboxylation and [3 + 2] cycloaddition take place in the ternary complex, whereas such a reactivity from bulky adamantane **3** is less likely. This proof of principle that decarboxylation and cycloaddition can be performed in the β-CD cavity has a significant importance for the design of new supramolecular systems for the control of photoreactivity.

## Introduction

Cycloadditions are highly useful reactions in organic synthesis providing complex cyclic structures from easily available precursors [1–2]. Among different reactions, [3 + 2] cycloadditions showed applicability in the synthesis of heterocyclic 5-ring compounds [3], as well as in the green synthesis of a number of natural products [4]. One of the useful synthons in [3 + 2] cycloadditions is azomethine ylide [5–7], also used in intramolecular reactions [8]. Azomethine ylides can be formed by several photochemical or thermal catalytic methods [5–7], including photodecarboxylation of phthalimide derivatives of α-amino acids such as *N*-phthaloylglycine (**1**) [9–10].

Phthalimide is a versatile chromophore that has been used in the synthesis of complex molecules and natural products [11] since the pioneering work of Kanaoka et al. [12]. Photochemical reactions of phthalimides include H-abstractions, cycloadditions and photoinduced electron transfer (PET)[13]. We became interested in the application of photochemical H-abstraction reactions initiated by phthalimides in organic synthesis [14–15]. Furthermore, H-abstractions were investigated in inclusion complexes, in the cavity of β-cyclodextrins (β-CD) [16]. We found out that H-abstraction reactions were about ten times more efficient in the β-CD complexes than in the isotropic solution, and the macrocyclic host affected the stereochemistry of the reaction. Moreover, we studied photodecarboxylation reactions initiated by the phthalimide chromophore [17–19] and applied them in cyclizations with memory of chirality [20] and diastereoselective peptide cyclizations [21]. Photodecarboxylations were also intensively investigated in a series of nonsteroidal anti-inflammatory drugs [22–24] such as ketoprofen [25–34], due to photoallergic responses initiated by photodecarboxylation of these drugs [35].

**Figure 1:**
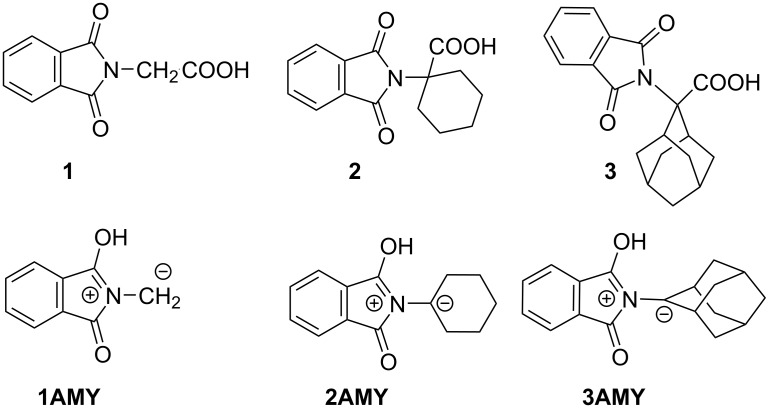
Phthalimide derivatives **1**–**3** and the corresponding azomethine ylides **1AMY**-**3AMY**.

Stereoselectivity in photochemical reactions can be achieved by use of supramolecular chemistry [36–37]. For example, stereoselectivity has been reported for photochemical reactions taking place in the inclusion complexes with CD [38–41] or structurally modified CDs [42–47]. Since β-CD is often used in pharmaceutical applications for solubilization of drugs or drug delivery [48], it would be interesting to investigate its effects to the photodecarboxylation reaction. Therefore, we investigated photochemical reactivity of phthalimide derivatives **1**–**3** (Figure 1) in solution without β-CD and in the β-CD inclusion complexes. Phthalimides **1**–**3** yield azomethine ylides **1AMY**-**3AMY** that are anticipated to react with acrylonitrile (AN) in [3 + 2] cycloadditions, which should be affected by β-CD.

## Results and Discussion

Phthalimide derivatives **1**–**3** were prepared according to procedures published in precedent literature [17]. The synthesis involves condensation of phthalic anhydride with unprotected amino acid. The synthetic procedures and characterization of compounds are reported in Supporting Information File 1. Irradiation of **1** was conducted first in CH_3_CN in the presence of AN, with or without addition of H_2_O (Table 1). Phthalimide **1** most probably undergoes decarboxylation delivering **1AMY** from the S_1_ state [49]. In CH_3_CN, **1AMY** decays with a rate constant of 2.9 × 10^6^ M^−1^ s^−1^, and reacts with methyl acrylate in [3 + 2] cycloaddition with the rate constant 2.7 × 10^7^ M^−1^ s^−1^ [49]. Protic solvents such as CH_3_OH or H_2_O quench azomethyine ylides, giving formal 1,4 H-shifted products. Thus, in the presence of H_2_O, no cycloaddition products are anticipated. However, it should be probed if the [3 + 2] cycloaddition can compete with the 1,4 H-shift upon irradiation of an inclusion complex.

**Scheme 1:**
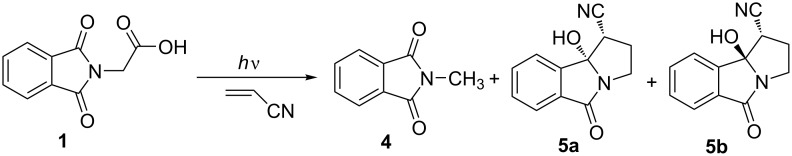
Irradiation of **1** in the presence of acrylonitrile (AN).

Under our conditions, photodecarboxylation of **1** was very inefficient in aprotic solvents, and it gave a mixture of simple decarboxylation product **4**, formed from **1AMY** by 1,4-H shift, in addition to the cycloadducts **5a** and **5b** (Scheme 1). Only use of a strong irradiation source such as a high pressure Hg lamp (400 W) provided higher yields of the cycloadducts. On the other hand, upon photolysis in the presence of H_2_O and a base, the photoreaction is about twenty times more efficient, but it delivers simple decarboxylation product **4** only. Attempts to use β-CD to complex both reactants and enhance the efficiency for the cycloaddition failed. In the photolysis of **1**, β-CD had no effect (Table 1), which may be ascribed to a small size of **1** that cannot fit well in the large cavity of β-CD and form a stable complex.

**Table 1:** Irradiation conditions, conversions and product ratio for photolysis of **1**.^a^

irradiation conditions	solvent/ irradiation time	conversion (%)	product ratio^b^

300 nm; *c*(**1**) = 10 mM, *c*(AN) = 0.25 M	CH_3_CN 18 h	4	**4**/**5a**/**5b** = 1:1:1
300 nm, *c*(**1**) = 10 mM, *c*(AN) = 0.25 M	CH_3_CN/H_2_O (1:3) 18 h	100	**4**/**5a**/**5b** = 1:0:0
Hg-HP, *c*(**1**) = 10 mM, *c*(AN) = 0.10 M	CH_3_CN 18 h	42	**4**/**5a**/**5b** = 2:1:1
300 nm, *c*(**1**) = 0.8 mM, *c*(AN) = 0.64 M	CH_3_CN/H_2_O (1:3) 1 h	97	**4**/**5a**/**5b** = 1:0:0
300 nm, *c*(**1**) = 0.8 mM, *c*(AN) = 0.25 M, *c*(β-CD) = 0.08 mM	CH_3_CN/H_2_O (1:3) 1 h	96	**4/5a/5b** = 1:0:0

^a^Irradiations were conducted in CH_3_CN, or CH_3_CN-H_2_O (3:1 v/v) in the presence of a base K_2_CO_3_ to deprotonate the acid. Irradiated in a Rayonet reactor using 12 lamps (1 lamp – 8 W) with the output at 300 nm, or by use of a high pressure mercury lamp (400 W Hg-HP). ^b^The product ratio determined by NMR.

**Figure 2:**
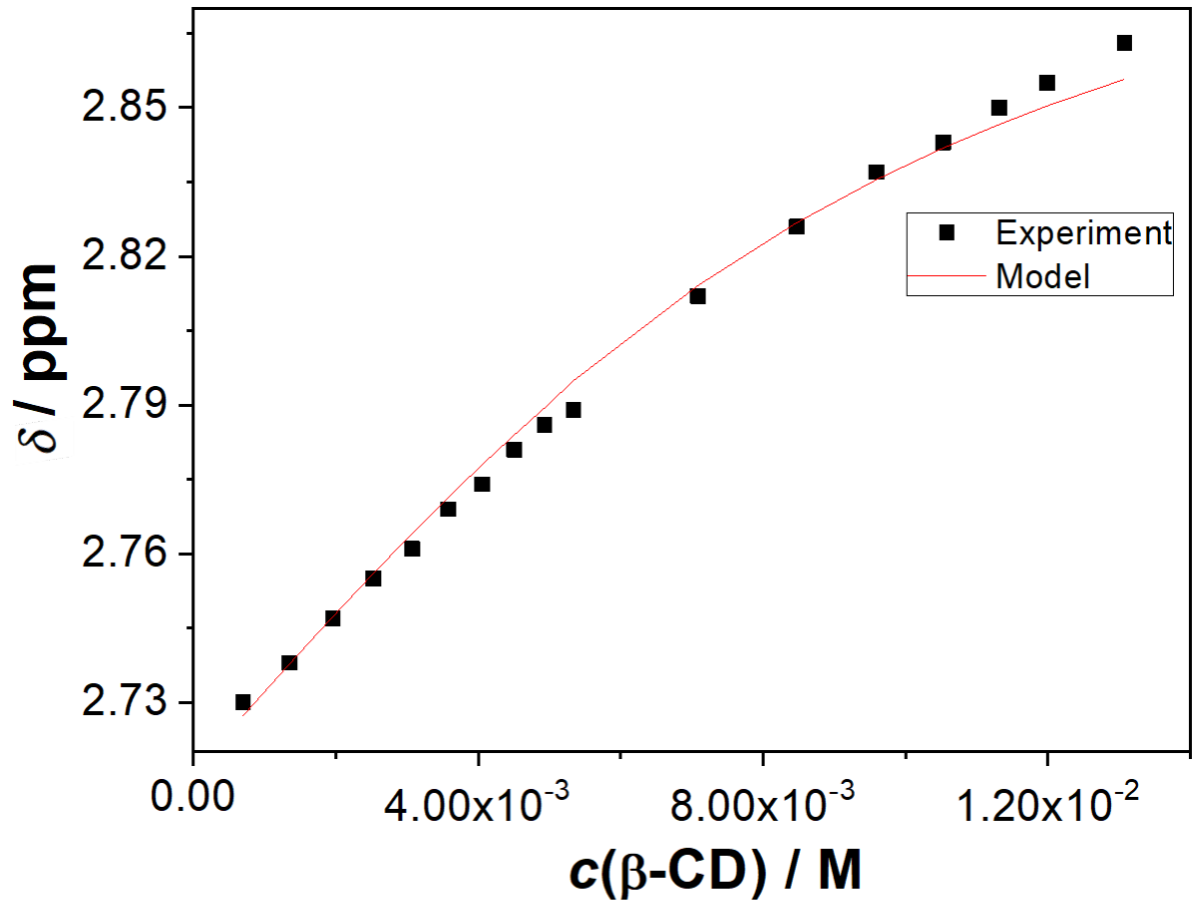
Dependence of the chemical shift of the H-atom at the cyclohexane 2 position in compound **2** on the β-CD concentration. Dots are experimental values and the red line corresponds to the calculated values by the WINEQNMR program [51] according to the model for the formation of 1:1 stoichiometry of the inclusion complex **2@β-CD**.

Molecules **2** and **3** are larger, and anticipated to form more stable inclusion complexes with β-CD. Therefore, we performed ^1^H NMR titrations to determine stability constants for the inclusion complexes **2@β-CD** and **3@β-CD**. The NMR titration for **2** with β-CD was carried out in CD_3_CN/D_2_O (3:7 v/v). The addition of β-CD to the solution of **2** induced downfield shifts of the H-signals corresponding to the cyclohexane 2 and 6 positions, as well as H-signals of the phthalimide, which changed from a singlet to a multiplet (Figure S1 in Supporting Information File 1). The spectral changes are in accordance with the formation of an inclusion complex **2@β-CD**, with the dynamics for the complexation faster than the NMR time-scale (millisecond). Nonlinear regression analysis of the chemical shifts depending on the β-CD concentration, to a complexation model with 1:1 stoichiometry, showed good correlation (Figure 2 and Figure S2 in Supporting Information File 1), with the stability constant for **2@β-CD** *K* = 190 ± 50 M^−1^. Formation of the inclusion complex was also confirmed by a NOESY spectrum where an interaction between the phthalimide H-atoms and the β-CD was observed (Figure S3 in Supporting Information File 1). Note that a compound similar to **2**, but with an amino instead of the carboxylic functional group, forms an almost 35 times more stable complex with β-CD (4-amino-*N*-cyclohexylphthalimide, *K* = 6200 M^−1^) [50].

An analogous titration in CD_3_CN/D_2_O (3:7 v/v) was also conducted for **3** with β-CD. The addition of β-CD to the solution of **3** induced downfield shifts of the signal corresponding to the adamantane 6 position and the phthalimide signals, which changed from singlet to a multiplet (Figure S4 in Supporting Information File 1). Nonlinear regression analysis of the chemical shifts to the β-CD concentration did not provide a good quality of the fit to the model involving 1:1 complex formation (Figure S5 in Supporting Information File 1). However, the approximated association constant for **3@β-CD**, *K* = 2600 ± 600 M^−1^, is similar to the known association constants for different adamantane derivatives with β-CD (*K* = 10^3^–10^5^ M^−1^) [52], in agreement with the anticipated good fit of the adamantane moiety in **3** to the β-CD cavity.

**Scheme 2:**
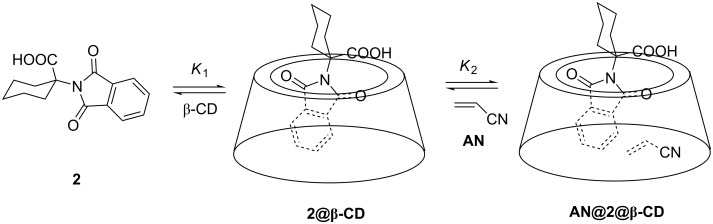
Complexation of **2** with β-CD, and formation of a ternary complex **AN@2@β-CD**.

After demonstrating the formation of inclusion complexes **2@β-CD** and **3@β-CD**, we investigated the possibility for the formation of ternary complexes with AN. Therefore, we titrated solutions of **2** or **3** with AN. The solutions of **2** or **3** contained a high concentration of β-CD to assure that the phthalimide derivative was in the inclusion complex **2@β-CD** or **3@β-CD**, respectively. The addition of AN to the CD_3_CN/D_2_O (3:7 v/v) solution of **2@β-CD** induced changes in the spectra, opposite to those observed upon formation of **2@β-CD** (compare Figures S1 and S6 in Supporting Information File 1). The spectral changes are in accordance with the formation of a ternary complex **AN@2@β-CD** (Scheme 2). However, they may also indicate that excess of AN added to the solution competitively binds to β-CD forming a complex **AN@β-CD** and inducing dissociation of the **2@β-CD**. If we assume a model for the complex formation in the stoichiometry 1:1:1, the nonlinear regression analysis of the chemical shift changes depending on the AN concentration and provided a poor fit with the estimated *K*_2_ value of 0–6 M^−1^ (Figure S7 in Supporting Information File 1).

We investigated also the possibility for the formation of the ternary complex **AN@3@β-CD**. Therefore, we titrated the solution of **3@β-CD** in CD_3_CN/D_2_O (3:7 v/v) with AN, whereupon spectral changes were observed (Figure S8 in Supporting Information File 1). The signal of the adamantane H-atom at the adamantane position 6 experienced a downfield shift, whereas the phthalimide signals experienced an upfield shifts. Although the changes were small, we tried to process them using nonlinear regression analysis and model for the complex formation with 1:1:1 stoichiometry. The fit was of poor quality, but it provided an estimation of the constant with the value of *K*_2_ = 0–7 M^−1^ (Figure S9 in Supporting Information File 1).

**Scheme 3:**
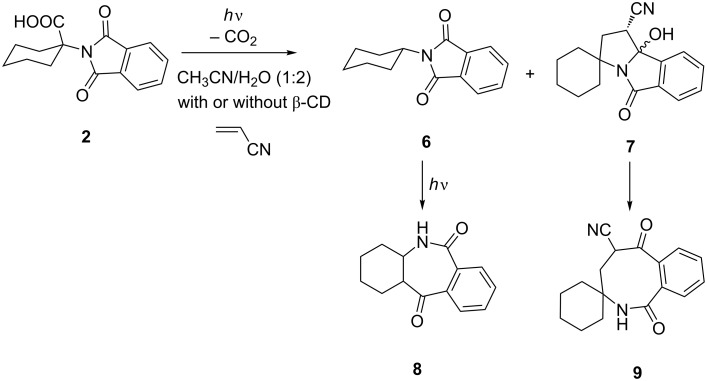
Photochemistry of **2** in the presence of AN, with or without β-CD.

**Scheme 4:**
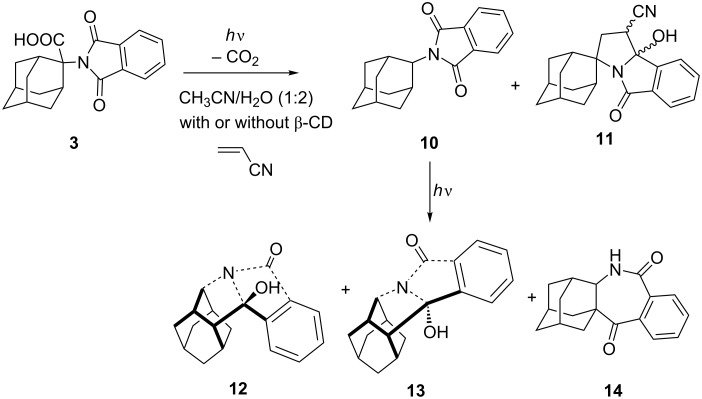
Photochemistry of **3** in the presence of AN, with or without β-CD.

The NMR titrations did not provide a clear evidence that ternary complexes were formed. However, formation of ternary complexes should affect the photochemical reactivity of **2** and **3** and cycloadditions of the corresponding azomethine ylides with AN. Therefore, we performed irradiation of solutions containing **2** and AN, **3** and AN, or the corresponding complexes **2@β-CD** and **3@β-CD** with AN. Scheme 3 and Scheme 4 show products formed in the photochemical reactions, whereas ratio of photoproducts obtained is given in Table 2.

**Table 2:** Irradiation conditions, conversions and product ratio after photolysis of **2** or **3**.^a^

irradiation conditions	solvent	conversion (%) /unidentified products (%)	product ratio^b^

**2**	CH_3_CN	99/46	**6**/**7**/**8**/**9** = 25:0:1:0
**2**	CH_3_CN/H_2_O (1:1)	99/70	**6**/**7**/**8**/**9** = 2.6:0:1:0
**2**, *c*(AN) = 0.78 M	CH_3_CN	45/40	**6**/**7**/**8**/**9** = 2:1:2:0
**2**, *c*(AN) = 0.78 M	CH_3_CN/H_2_O (1:1)	99/61	**6**/**7**/**8**/**9** = 1.5:0:1:0
**2**, *c*(β-CD) = 22 mM	CH_3_CN/H_2_O (1:1)	99/69	**6**/**7**/**8**/**9** = 4:0:1:0
**2**, *c*(AN) = 0.78 M, *c*(β-CD) = 22 mM	CH_3_CN/H_2_O (1:1)	58/36	**6**/**7**/**8**/**9** = 4.5:1:3:2.5
**3**	CH_3_CN	10/7	**10**/**11**/**12**/**13**/**14** = 1:0:0:0:0
**3**	CH_3_CN/H_2_O (1:1)	52/50	**10**/**11**/**12**/**13**/**14** = 1:0:0:0:0
**3**, *c*(K_2_CO_3_) = 0.7 mM	CH_3_CN/H_2_O (1:1)	55/5	**10**/**11**/**12**/**13**/**14** = 1:0:0:0:0
**3**, *c*(AN) = 0.74 M,	CH_3_CN	33/2	**10**/**11**/**12**/**13**/**14** = 1:5:9:8:3
**3**, *c*(AN) = 0.74 M,	CH_3_CN/H_2_O (1:1)	75/56	**10**/**11**/**12**/**13**/**14** = 1:5:5:2:1
**3**, *c*(β-CD) = 15 mM	CH_3_CN/H_2_O (1:1)	58/40	**10**/**11**/**12**/**13**/**14** = 1:0:5:1:0
**3**, *c*(AN) = 0.74 M, *c*(β-CD) = 15 mM	CH_3_CN/H_2_O (1:1)	45/21	**10**/**11**/**12**/**13**/**14** = 1:1:5:2:1

^a^Irradiations of **2** (*c* = 2.0 mM) or **3** (*c* = 1.4 mM) were conducted in CH_3_CN, or CH_3_CN/H_2_O (1:1 v/v), with or without acrylonitrile (AN) and β-CD. All samples were irradiated in a Luzchem reactor using 8 lamps (1 lamp – 8 W) with the output at 300 nm for 30 min (compound **2**) or 35 min (compound **3**). The detailed procedure can be found in the experimental part. ^b^The product ratio was determined by HPLC–MS and NMR.

The addition of H_2_O to the solution of **2** or **3** in CH_3_CN generally increase the efficiency of the decarboxylation reaction, as well as the efficiency of the secondary photochemical H-abstraction from primarily formed products **6** or **10**, giving **8**, or **12–14**, respectively. Upon addition of AN to the solution of **2** or **3** in CH_3_CN, in aprotic conditions, **2AMY** and **3AMY** should be formed and intercepted with AN to yield cycloadducts **7** or **11**, respectively. However, the formation of cycloadducts is very inefficient, which may be ascribed to a smaller rate constant for the quenching due to steric hindrance imposed by the bulky cyclohexane or adamantine moiety. Thus, irradiation of **2** gave cycloadducts **7** in ≈9% yield, and **3** gave cycloadducts **11** in ≈6% yield. Furthermore, it is anticipated that the addition of H_2_O quenches the cycloaddition reaction due to a faster reaction of **AMY** with H_2_O then with AN, giving formal 1,4-H-shifted products. Indeed, H_2_O quenched the cycloaddition of **2AMY** with AN, but it did not quench the cycloaddition of **3AMY** with AN. Thus, cycloadduct **11** was detected after irradiation of **3** with AN in CH_3_CN/H_2_O, but not when a base was added to the solution.

Addition of β-CD did not affect the decarboxylation reaction, since the same conversion to photoproducts was observed in the presence and absence of β-CD. However, for the adamantane derivative in the presence of β-CD, the secondary photochemical H-abstraction became more efficient, resulting in a different product distribution. More efficient H-abstraction reaction in the presence of β-CD have been reported [16].

β-CD affected the cycloaddition reaction of photogenerated AMY with AN. Upon irradiation of **2** with AN in the presence of β-CD, the cycloadduct was formed in ≈5% yield, even though the irradiation was conducted in aqueous solution. The finding suggests that formation of a ternary complex **AN@2@β-CD** is possible and that photolysis of **2** in such a complex yields **2AMY**, which is then readily intercepted with AN in the same complex. Note that cycloadduct **11** was also detected (≈4%), upon irradiation of **3** with AN in the presence of β-CD, suggesting that the photodecarboxylation reaction and subsequent [3 + 2] cycloaddition take place in the ternary complex **AN@3@β-CD**. However, cycloadduct **11** was formed in a higher yield (≈20%) when **3** was photolyzed with AN in the aqueous solution without β-CD. Although a reason for the different effect of β-CD is not clear, it may be due to a lower stability of the ternary complex **AN@3@β-CD**, compared to **AN@2@β-CD**. Namely, the adamantane is a bulky moiety that occupies most of the space in the inclusion complex **3@β-CD**, making formation of the ternary complex less likely. Furthermore, if **AN@3@β-CD** was formed, photogenerated **3AMY@β-CD** may not be in the right orientation for the cycloaddition to take place, leading predominantly to the reaction of **3AMY** with H_2_O.

## Conclusion

Herein we have demonstrated a proof of principle that β-CD can be used as a molecular container in which two molecules can be complexed, a phthalimide derivative and acrylonitrile, forming a ternary complex. Irradiation of such a complex leads to decarboxylation and formation of the reactive intermediate, azomethine ylide, within the supramolecular host. The subsequent [3 + 2] cycloaddition within the inclusion complex gives heterocyclic cycloadducts, even though it is conducted in aqueous solvent in which ylides have short lifetimes. The reaction needs to be optimized for different substrates with the right choice of host size. However, the proof of principle provides a new idea for the development of supramolecular systems for the tuning of photochemical reactivity.

## Experimental

### General

^1^H and ^13^C NMR spectroscopic data were recorded at room temperature on a Bruker Avance 300 MHz or Bruker Avance 600 MHz spectrometer. CD_3_OD or CD_3_CN-D_2_O were used as deuterated solvent. TMS (^1^H NMR) or deuterated solvent itself (^13^C NMR) was used as internal reference. Chemical shifts were reported in ppm. Irradiation experiments were performed in a Rayonet RPR-100 photoreactor equipped with 12 lamps or a Luzchem reactor equipped with 8 lamps with the maximum output at ≈300 nm (1 lamp – 8 W). During the irradiations in the Rayonet reactor, the irradiated solutions were continuously purged with Ar and cooled by a tap water finger condenser. Solvents for the irradiations were of HPLC purity. Chemicals were purchased from the usual commercial sources and used as received. Solvents for chromatographic separations were used from the supplier (p.a. or HPLC grade) as is or were purified by distillation (CH_2_Cl_2_). Semipreparative HPLC separations were performed on a Varian Pro Star instrument equipped with a Phenomenex Jupiter C18 5μ 300A column, using CH_3_OH/H_2_O + TFA as eluent. HPLC–MS analyses were conducted on an Agilent 1200 Series machine equipped with a DAD detector and a mass spectrometer with a triple quadrupole Agilent 6420 device.

#### Photochemistry of **2** and **3** under different conditions

A solution of **2** (30 mg, 0.11 mmol) in CH_3_CN (27.5 mL) was prepared and transferred to 7 quartz cuvettes (3.9 mL into each). Then, CH_3_CN (3.9 mL) or H_2_O (3.9 mL) was added to each of the cuvettes, followed by the addition of β-CD (200 mg, 0.176 mmol), acrylonitrile (AN, 0.4 mL, 6.1 mmol) or nothing (see Table 2). Solutions were purged with N_2_ for 15 min, sealed and irradiated at the same time in a Luzchem reactor at 300 nm (8 lamps) for 30 min. After the irradiation, solutions were extracted with EtOAc (3 × 3 mL), the extracts were dried over MgSO_4_, filtered and the solvent was removed on a rotary evaporator. The crude reaction mixtures were filtered through a plug of silica gel by use of CH_2_Cl_2_/EtOAc as eluent and were analyzed by ^1^H NMR and HPLC–MS (Table 2).

Alternatively, a solution of **3** (44 mg, 0.135 mmol) in CH_3_CN (50 mL) was prepared and transferred to 7 quartz cuvettes (7.0 mL into each). Then, CH_3_CN (7.0 mL) or H_2_O (7.0 mL) was added to each of the cuvettes, followed by the addition of β-CD (240 mg, 0.21 mmol), AN (0.7 mL, 10.7 mmol), K_2_CO_3_ (1.3 mg, 0.009 mmol), or nothing (see Table 2). Solutions were purged with N_2_ for 15 min, sealed and irradiated at the same time in a Luzchem reactor at 300 nm (8 lamps) for 35 min. After the above described work up, the composition of the irradiated solutions was analyzed by ^1^H NMR and HPLC–MS (Table 2).

#### Preparative irradiation of **2** with AN and with β-CD

Phthalimide **2** (110 mg, 0.403 mmol) was dissolved in CH_3_CN (50 mL) and this solution was added slowly to the solution of β-CD (4.0 g, 3.52 mmol) in H_2_O (250 mL). The solution was sonicated for 15 min and then AN (10 mL, 152.6 mmol) was added. After sonicating for additional 15 min, the solution was transferred to fifteen quartz test tubes (each containing 20 mL), purged with N_2_ for 20 min and sealed. The solutions were irradiated for 1 h in a Luzchem reactor using 8 lamps with the output at 300 nm. When the irradiation was completed, the irradiated solutions were combined and extracted with pentane (3 × 50 mL), and then with CH_2_Cl_2_ (2 × 50 mL) and EtOAc (2 × 50 mL). The organic extracts were dried over anhydrous Na_2_SO_4_, and CH_2_Cl_2_ and EtOAc were combined. The solutions were filtered and the solvent was removed on a rotary evaporator. The photoproducts were separated by chromatography on semipreparative HPLC followed by preparative TLC using 5% MeOH/10% Et_2_O/85% CH_2_Cl_2_ and 40% EtOAc/CH_2_Cl_2_ as eluent. Compound **6** (11 mg, 10%) was identified by comparison of the spectra with those from precedent literature [53].

HPLC method: 0–10 min (25% H_2_O/MeOH), 10–30 min (25–0% H_2_O/MeOH), 30–40 min (MeOH), 40-45 min (0–25% H_2_O/MeOH).

**9b'-Hydroxy-5'-oxo-1',2',5',9b'-tetrahydrospiro[cyclohexane-1,3'-pyrrolo[2,1-*a*]isoindole]-1'-carbonitrile (7):** 2 mg (2%), oily crystals; ^1^H NMR (CD_3_OD, 600 MHz) δ 7.81 (dd, *J* = 1.0, 7.6 Hz, 1H), 7.70 (dt, *J* = 1.3, 7.6 Hz, 1H), 7.65 (dt, *J* = 1.3, 7.6 Hz, 1H), 7.48 (dd, *J* = 1.0, 7.6 Hz, 1H), 4.55 (br s, 3H), 3.48–3.42 (m, 1H), 2.65–2.59 (m, 1H), 2.06–2.00 (m, 2H), 1.86–1.78 (m, 2H), 1.53–1.27 (m, 7H); MS *m*/*z* (% relative intensity): 282 (100), 283 (18.4), 284 (1.6).

**1,3,4,4a,5,11a-Hexahydro-6*H*-dibenzo[*b*,*e*]azepine-6,11(2*H*)-dione (8):** 4 mg (4%), oily crystals; ^1^H NMR (CD_3_OD, 300 MHz) δ 7.83–7.77 (m, 1H), 7.71–7.61 (m, 2H), 7.55–7.49 (m, 1H), 4.18 (d, *J* =2.4 Hz, 1H), 2.74–2.64 (m, 1H), 2.31–2.20 (m, 1H), 1.97–1.84 (m, 5H), 1.47–1.34 (m, 1H); ^13^C NMR (CD_3_OD, 75 MHz) δ 207.1 (s, 1C), 172.2 (s, 1C), 139.1 (s, 1C), 133.3 (s, 1C), 133.2 (d, 1C), 133.1 (d, 1C), 130.0 (d, 1C), 128.8 (d, 1C), 57.3 (d, 1C), 49.7 (d, 1C), 30.1 (t, 1C), 26.2 (t, 1C), 23.7 (t, 1C), 21.3 (t, 1C); MS *m*/*z* (% relative intensity): 229 (100), 230 (15.1), 231 (1.1).

**1,6-Dioxo-1,4,5,6-tetrahydro-2*H*-spiro[benzo[*c*]azocine-3,1'-cyclohexane]-5-carbonitrile (9):** 3 mg (3%), oily crystals; ^1^H NMR (CD_3_OD, 300 MHz) δ 8.19-8.15 (m, 1H), 8.04–8.00 (m, 1H), 7.80–7.66 (m, 2H), 2.84 (dd, *J* = 3.3, 12.0 Hz, 1H), 2.50–2.30 (m, 2H), 2.10–1.70 (m, 8H), 1.55–1.35 (m, 2H); ^13^C NMR (CD_3_OD, 75 MHz) δ 202.5 (s, 1C), 134.9 (s, 1C), 134.7 (d, 1C), 133.4 (d, 1C), 132.9 (d, 1C), 130.6 (d, 1C), 120.7 (s, 1C, CN), 62.0 (d, 1C), 36.1 (t, 1C), 35.7 (t, 1C), 30.7 (s, 1C), 28.0 (t, 1C), 26.5 (t, 1C), 21.7 (t, 1C), 11.7 (t, 1C), signals for 2 quaternary C-atoms were not observed; MS *m*/*z* (% relative intensity): 282 (100), 283 (18.4), 284 (1.6).

#### Preparative irradiation of phthalimide **3** with AN and with β-CD

Phthalimide **3** (150 mg, 0.461 mmol) was dissolved in CH_3_CN (100 mL) and this solution was added slowly to the solution of β-CD (5.23 g, 4.61 mmol) in H_2_O (370 mL). The solution was sonicated for 15 min and then AN (10 mL, 152.6 mmol) was added. After sonicating for additional 15 min, the solution was transferred to twenty quartz test tubes (each containing ≈20 mL), purged with N_2_ for 20 min and sealed. The solutions were irradiated for 4 h in a Luzchem reactor using 8 lamps with the output at 300 nm. When the irradiation was completed, the irradiated solutions were combined and extracted with pentane (3 × 50 mL), and then with CH_2_Cl_2_ (2 × 50 mL) and EtOAc (2 × 50 mL). The organic extracts were dried over anhydrous Na_2_SO_4_, and CH_2_Cl_2_ and EtOAc were combined. The solutions were filtered and the solvent was removed on a rotary evaporator. The photoproducts were separated by chromatography on a column of silica gel using 2–10% MeOH/CH_2_Cl_2_ followed by preparative TLC with 5% MeOH/DCM and 5% MeOH/10% Et_2_O/DCM. The separation afforded only products **10**, **12**, **13**, and **14**, which were identified by comparison of the spectra with literature precedent [54].

#### Preparative irradiation of phthalimide **3** with AN and without β-CD

A solution of phthalimide **3** (142 mg, 0.450 mmol) and AN (5 mL, 76.33 mmol) in CH_3_CN (100 mL) was poured to a quartz Erlenmayer flask. The solution was purged with N_2_ for 30 min and then irradiated for 20 h with continuous stirring. After the irradiation, the solvent was removed on a rotary evaporator and the crude reaction mixture was chromatographed on a column of SiO_2_ with 0–10% MeOH/CH_2_Cl_2_ as eluent, followed by chromatography on a semipreparative HPLC with 0–50% H_2_O/MeOH, 0.1% TFA as eluent, and finally by preparative TLC with 3% MeOH/CH_2_Cl_2_ as eluent.

HPLC method: 0–5 min (35% H_2_O/MeOH, 0.1% TFA), 5–20 min (35–0% H_2_O/MeOH, 0.1% TFA), 20–25 min (MeOH), 25–30 min (0−35% H_2_O/MeOH, 0.1% TFA).

**9b'-Methyl-5'-oxo-1',2',5',9b'-tetrahydrospiro[adamantane-2,3'-pyrrolo[2,1-*a*]isoindole]-1'-carbonitrile (11):** 2 mg (2%), oily crystals; ^1^H NMR (CD_3_OD, 300 MHz) δ 7.70–7.65 (m, 2H), 7.59 (d, *J* = 7.6 Hz, 1H), 7.53 (dt, *J* = 0.6, 7.4 Hz, 1H), 4.03 (d, *J* = 7.9 Hz, 1H), 2.30–2.26 (m, 1H), 2.04–2.00 (m, 1H), 1.97–1.91 (m, 5H), 1.89–1.85 (m, 3H), 1.80–1.76 (m, 1H); ^13^C NMR (CD_3_OD, 75 MHz) δ 208.6 (s, 1C), 135.4 (s, 1C), 134.7 (d, 1C), 132.9 (s, 1C), 130.8 (d, 1C), 124.2 (d, 1C), 123.3 (d, 1C), 98.1 (s, 1C), 71.6 (s, 1C), 39.0 (t, 1C), 37.9 (t, 1C), 35.5 (t, 1C), 35.3 (d, 1C), 34.3 (d, 1C), 33.5 (t, 1C), 33.2 (t, 1C), 30.7 (d, 1C), 28.6 (d, 1C), 28.3 (d, 1C), 27.3 (t, 1C), the singlet corresponding to the CN was not observed; MS *m*/*z* (% relative intensity): 334 (100), 335 (22.7), 336 (2.5).

#### NMR titrations with β-CD

A solution of **2** (*c* = 7.21 mM), or **3** (*c* = 2.83 mM) in CD_3_CN/D_2_O (3:7 v/v, 1.0 or 0.5 mL, respectively) in NMR tube was titrated with a solution of β-CD (*c* = 20.5 mM). After each addition of β-CD, an ^1^H NMR spectrum was recorded. The changes of chemical shifts depending on the β-CD concentration were processed by nonlinear regression analysis using WinEQNMR software [51]. The titration was performed at 25 °C.

#### NMR titrations with AN

A solution of **2@β-CD** (prepared by mixing **2** in the concentration of 2.71 mM with β-CD in the concentration of 8.20 mM), or **3@β-CD** (prepared by mixing **3** in the concentration of 1.54 mM with β-CD in the concentration of 20.5 mM) in CD_3_CN/D_2_O (3:7 v/v, 1.0 mL) was titrated with acrylonitrile (AN). After each addition of AN, an ^1^H NMR spectrum was recorded. The changes of chemical shifts depending on the AN concentration were processed by nonlinear regression analysis using WinEQNMR software [51]. The titration was performed at 25 °C.

## Supporting Information

File 1 Experimental procedures, characterization of the known compounds, NMR spectra from the titration experiments and copies of ^1^H and ^13^C NMR spectra of all compounds.
