# Supplementary material for: [3 + 2] Cycloaddition with photogenerated azomethine ylides in β-cyclodextrin
Source: Beilstein J Org Chem. 2020 Jun 12;16:1296–304. doi: 10.3762/bjoc.16.110 (PMC7296198; doi:10.3762/bjoc.16.110)
Supplement: File 1 — Experimental procedures, characterization of the known compounds, NMR spectra from the titration experiments and copies of 1H and 13C NMR spectra of all compounds. [file Beilstein_J_Org_Chem-16-1296-s001.pdf]

BEILSTEIN JOURNAL OF ORGANIC CHEMISTRY

Supporting Information

for

[3 + 2] Cycloaddition with photogenerated azomethine ylides in β-cyclodextrin

Margareta Sohora, Leo Mandić and Nikola Basarić

Beilstein J. Org. Chem. **2020,** *16,* 1296–1304. doi:10.3762/bjoc.16.110

Experimental procedures, characterization of the known compounds, NMR spectra from the titration experiments and copies of ^{1}H and ^{13}C NMR spectra of all compounds

License and Terms: This is a supporting information file under the terms of the Creative Commons Attribution License (http://creativecommons.org/licenses/by/4.0). Please note that the reuse, redistribution and reproduction in particular requires that the authors and source are credited.
The license is subject to the *Beilstein Journal of Organic Chemistry* terms and conditions: (https://www.beilstein-journals.org/bjoc)

Content:

1. Experimental procedures and synthesis of known compounds S2

2. Determination of K_a values with β-CD by NMR titrations (Figures S1–S9 and Table S1) S6

3. NMR spectra S15

4. References S28

1. Experimental procedures and synthesis of known compounds

Synthesis of phthalimides 1 and 2

A flask (50 mL) was charged with phthalic anhydride (10 mmol), which was melted. To the melt, amino acid (5 mmol) was added in several portions. The reaction mixture was heated at 170–180 °C for 15 min with a stopper, and 15 min without. To the cooled reaction mixture CH_2Cl_2 (100 mL) was added and it was washed with aqueous HCl (1 M, 2 × 20 mL). The organic solution was dried over anhydrous $MgSO_4$, filtered and the solvent was removed on a rotary evaporator. The residue was chromatographed on silica gel column using CH_2Cl_2/EtOAc (0 → 20%) as eluent.

***N*-phthaloylglycine (1) [[1]]**

Prepared according to the general procedure from glycine (2.50 g, 33.4 mmol) and phthalic anhydride (5.02 g, 33.9 mmol). The pure product (4.84 g, 71%) was obtained in the form of colorless crystals.

^{1}H NMR ($CDCl_3$, 600 MHz) δ/ppm: 7.87-7.93 (m, 2H), 7.72-7.78 (m, 2H), 4.49 (s, 2H); ^{13}C NMR ($CDCl_3$, 150 MHz) δ/ppm: 172.1 (s, 1C), 167.4 (s, 2C), 134.5 (d, 2C), 132.1 (s, 2C), 123.9 (d, 2C), 38.6 (t, 1C).

1-(*N*-phthalimido)cyclohexane-1-carboxylic acid (2) [[2]]

Prepared according to the general procedure from 1-aminocyclohexane-1-carboxylic acid (345 mg, 2.4 mmol) and phthalic anhydride (415 mg, 2.8 mmol). The pure product (105 mg, 16%) was obtained in the form of colorless crystals.

^{1}H NMR ($CDCl_3$, 300 MHz) δ/ppm: 7.76-7.84 (m, 2H), 7.66-7.74 (m, 2H), 2.86-3.00 (m, 2H), 2.01-2.15 (m, 2H), 1.67-1.83 (m, 2H), 1.46-1.66 (m, 4H), COOH signal was not observed; ^{13}C NMR ($CDCl_3$, 150 MHz) δ/ppm: 178.3 (s, 1C), 169.2 (s, 2C), 134.2 (d, 2C), 132.0 (s, 2C), 123.3 (d, 2C), 65.3 (s, 1C), 32.0 (t, 2C), 25.1 (t, 1C), 22.6 (t, 2C).

Synthesis of 2-(*N*-phthalimido)adamantane-2-carboxylic acid (3) [[3]]

A flask (50 mL) was charged with 2-aminoadamantane-2-carboxylic acid [[4]] (1.49 g, 7.6 mmol), phthalic anhydride (2.24 g, 15.1 mmol) and DMF (4 mL). The reaction mixture was stirred at 170–180 °C over 2–3 days. The solvent was removed on a rotary evaporator and to the residue CH_3CN

(50 mL) was added. Unreacted amino acid was filtered off on a sinter funnel and washed with additional CH_3CN (100 mL) and acetone (100 mL). The solvent from the combined mother liquor and organic solutions was removed on a rotary evaporator and the residue was chromatographed on silica gel column using CH_2Cl_2/EtOAc (0 → 20%) as eluent to afford the pure product (780 mg, 31%) obtained in the form of colorless crystals.

^{1}H NMR (DMSO-d_6, 300 MHz) δ/ppm: 12.87 (br s, 1H), 7.80-7.89 (m, 4H), 3.63 (br s, 2H), 2.10 (d, J = 12.2 Hz, 2H), 1.60-1.92 (m, 10H); ^{13}C NMR (DMSO-d_6, 75 MHz) δ/ppm: 171.8 (s, 1C), 169.0 (s, 2C), 134.7 (d, 2C), 131.1 (s, 2C), 122.8 (d, 2C), 71.2 (s, 1C), 36.9 (t, 1C), 33.8 (t, 2C), 33.5 (t, 2C), 29.6 (d, 2C), 25.7 (d, 1C), 25.6 (d, 1C).

Irradiation of phthalimide 1

A glass vessel equipped with an inlet for an Hg-lamp, condenser and inlet for N_2 was charged with a solution of **1** (1.56 g, 7.6 mmol) in CH_3CN (635 mL), to which acrylonitrile (5 mL, 4.05 g, 76.3 mmol) was added. The solution was purged with Ar for 20 min, and then irradiated with a high pressure Hg lamp (400 W) over 9 h, with continuous cooling by H_2O and purging with Ar. After the irradiation, the solvent was removed on a rotary evaporator and the residue was dissolved in EtOAc (100 mL), which was washed with aqueous $NaHCO_3$ (3 × 25 mL). The aqueous layer was washed with Et_2O (2 × 25 mL), and then acidified with aqueous conc. HCl until pH 3 was reached and extracted again with EtOAc (3 × 50 mL). All organic solutions were combined, dried over anhydrous $MgSO_4$, filtered and the solvent was removed on a rotary evaporator. The residue was chromatographed on a column of silica gel using CH_3OH/CH_2Cl_2 (0 → 5%), to afford *N*-methylphthalimide (**4**) [5] and a mixture of adducts **5** [6] (140 mg, 9%).

N-methylphthalimide (**4**) 73 mg (5 %); ^{1}H NMR ($CDCl_3$, 300 MHz) δ/ppm: 7.81-7.89 (m, 2H), 7.67-7.75 (m, 2H), 3.18 (s, 3H); ^{13}C NMR ($CDCl_3$, 75 MHz) δ/ppm: 168.6 (s, 2C), 134.0 (d, 2C), 132.4 (s, 2C), 123.3 (d, 2C), 24.0 (q, 1C).

Irradiation of 1 in the presence of β-CD

A glass vessel (200 mL) was filled with a solution of **1** (16.5 mg, 0.080 mmol), and acrylonitrile (4 mL, 61.1 mmol, 759 equiv) in CH_3CN (10 mL), to which a solution of β-CD (22 mg, 0.019 mmol) in H_2O (86 mL) was added. The solution was purged with Ar for 30 min and irradiated in a Rayonet reactor at 300 nm over 1 h. The irradiated solution was continuously purged with Ar

and cooled by a H_2O finger-condenser. After the irradiation, an extraction was carried out with EtOAc/CH_2Cl_2 (1:1) (4 × 50 mL). The combined extracts were dried over anhydrous $MgSO_4$, filtered and the solvent was removed on a rotary evaporator. Analysis of the irradiated mixture by ^{1}H NMR indicated formation of **4** as the only product in the yield of 97%.

Characterization of known photoproducts after photolysis of 2

***N*-cyclohexylphthalimide (6)** [7] ^{1}H NMR (300 MHz, $CDCl_3$) *δ*/ppm: 7.85-7.78 (m, 2H), 7.73-7.66 (m, 2H), 4.17-4.05 (m, 1H), 2.28-1.82 (m, 2H), 1.77-1.66 (m, 2H), 1.46-1.23 (m, 4H); ^{13}C NMR (75 MHz, $CDCl_3$) *δ*/ppm: 168.5 (s, 2C), 133.7 (d, 2C), 132.1 (s, 2C), 123.0 (d, 2C), 50.9 (d, 1C), 29.9 (t, 2C), 26.0 (t, 2C), 25.1 (t, 1C).

Characterization of known photoproducts after photolysis of 3

2-Adamantylphthalimide (10) [8] ^{1}H NMR (300 MHz, $CDCl_3$) *δ*/ppm: 7.80-7.77 (m, 2H), 7.69-7.66 (m, 2H), 4.32 (br s, 1H), 2.57 (br s, 2H), 2.33 (br s, 1H), 2.29 (br s, 1H), 1.96-1.92 (m, 6H), 1.79 (br s, 2H), 1.71-1.66 (m, 2H); ^{13}C NMR (75 MHz, $CDCl_3$) *δ*/ppm: 169.4 (s, 2C), 133.6 (d, 2C), 132.0 (s, 2C), 122.6 (d, 2C), 61.2 (d, 1C), 38.5 (t, 2C), 37.6 (t, 1C), 32.7 (t, 2C), 31.9 (d, 2C), 27.5 (d, 1C), 26.8 (d, 1C).

2-*exo*-Hydroxy-10-aza-hexacyclo[9.5.1.1^{12,14}.1^{16,18}.0^{2,10}.0^{3,8}]nonadeca-3,5,7-triene-9-one (12) [8] ^{1}H NMR ($CDCl_3$, 600 MHz) *δ*/ppm: 7.69 (d, *J* =7.5 Hz, 1H), 7.56 (ddd, *J* = 7.5, 7.3, 0.9 Hz, 1H), 7.49 (d, *J* = 7.5 Hz, 1H), 7.45 (ddd, *J* = 7.5, 7.3, 0.9 Hz, 1H), 4.04 (t, *J* = 4.8 Hz, 1H), 3.41 (br s, 1H, OH), 3.14 (br s, 1H), 1.99 (ddd, *J* = 13.2, 5.7, 2.7 Hz, 1H), 1.95 (ddd, *J* = 13.2, 5.7, 2.7 Hz, 1H), 1.90 (br s, 1H), 1.78 (ddd, *J* = 9.3, 6.2, 3.1 Hz, 1H), 1.72 (m, 1H), 1.63-1.67 (m, 2H), 1.04 (ddd, *J* = 14.0, 3.8, 2.0 Hz, 1H), 1.00 (d, 1H, *J* = 14.0 Hz, 1H); ^{13}C NMR ($CDCl_3$, 150 MHz) *δ*/ppm: 171.4 (s, 1C), 146.1 (s, 1C), 134.7 (s, 1C), 132.7 (d, 1C), 129.4 (d, 1C), 124.1 (d, 1C), 123.0 (d, 1C), 98.4 (s, 1C), 59.7 (d, 1C), 47.2 (d, 1C), 38.9 (d, 1C), 36.6 (t, 1C), 34.0 (t, 1C), 32.8 (t, 1C), 31.5 (t, 1C), 29.1 (d, 1C), 27.3 (d, 1C), 25.6 (d, 1C).

2-*endo*-Hydroxy-10-aza-hexacyclo[9.5.1.1^{12,14}.1^{16,18}.0^{2,10}.0^{3,8}]nonadeca-3,5,7-triene-9-one (13) [8] ^{1}H NMR ($CDCl_3$, 600 MHz) *δ*/ppm: 7.74 (dd, *J* = 7.6, 0.8 Hz, 1H), 7.62 (ddd, *J* = 7.4, 7.6, 1.0 Hz, 1H), 7.55 (d, *J* = 7.6 Hz, 1H), 7.47 (ddd, *J* = 7.4, 7.6, 0.9 Hz, 1H), 4.20 (t, *J* = 4.9 Hz, 1H),

2.80 (m, 1H), 2.69 (br s, 1H, OH), 2.60 (t, *J* = 3.9 Hz, 1H), 2.32 (br s, 1H), 2.26 (br s, 1H), 1.99 (ddd, *J* = 12.7, 5.7, 3.3 Hz, 1H), 1.93 (ddd, *J* = 12.9, 6.2, 3.3 Hz, 1H), 1.89 (br s, 1H), 1.80 (d, *J* = 12.7 Hz, 1H), 1.75-1.68 (m, 3H), 1.61-1.59 (m, 2H); ^{13}C NMR ($CDCl_3$, 150 MHz) δ/ppm: 179.2 (s, 1C), 151.2 (s, 1C), 133.7 (d, 1C), 132.7 (s, 1C), 129.6 (d, 1C), 123.9 (d, 1C), 122.4 (d, 1C), 98.6 (s, 1C), 67.3 (d, 1C), 46.5 (d, 1C), 38.9 (d, 1C), 36.1 (t, 1C), 35.3 (t, 1C), 34.6 (t, 1C), 32.7 (t, 1C), 32.1 (d, 1C), 28.5 (d, 1C), 25.3 (d, 1C).

10-Azapentacyclo[10.3.3.1^{14,16}.0^{1,11}.0^{3,8}]nonadeca-3,5,7-triene-2,9-dione (14) [8] ^{1}H NMR ($CDCl_3$, 600 MHz) δ/ppm: 7.91 (dd, *J* = 7.6, 1.2 Hz, 1H), 7.63 (dt, *J* = 7.5, 1.4 Hz, 1H), 7.58 (dt, *J* = 7.5, 1.4 Hz, 1H), 7.48 (dd, *J* = 7.6, 1.3 Hz, 1H), 6.55 (br s, 1H, NH), 3.88 (br s, 1H), 2.21 (ddd, *J* = 13.2, 4.5, 2.4 Hz, 1H), 2.12–2.09 (m, 2H), 2.06 (ddd, *J* = 13.3, 4.7, 2.5 Hz, 1H), 2.00 (br s, 1H), 1.90–1.82 (m, 4H), 1.80–1.76 (m, 1H), 1.73 (ddd, *J* = 13.3, 4.8, 2,3 Hz, 1H), 1.70–1.67 (m, 2H); ^{13}C NMR ($CDCl_3$, 150 MHz) δ/ppm: 208.4 (s, 1C), 170.0 (s, 1C), 137.0 (s, 2C), 131.8 (d, 2C), 131.7 (s, 1C), 129.2 (d, 1C), 128.2 (d, 1C), 56.6 (d, 1C), 53.2 (s, 1C), 39.3 (t, 1C), 36.4 (t, 1C), 36.2 (t, 1C), 31.2 (d, 1C), 31.1 (t, 1C), 30.6 (t, 1C), 26.8 (d, 1C), 26.7 (d, 1C).

2. Determination of K_a values with β-CD by NMR titrations

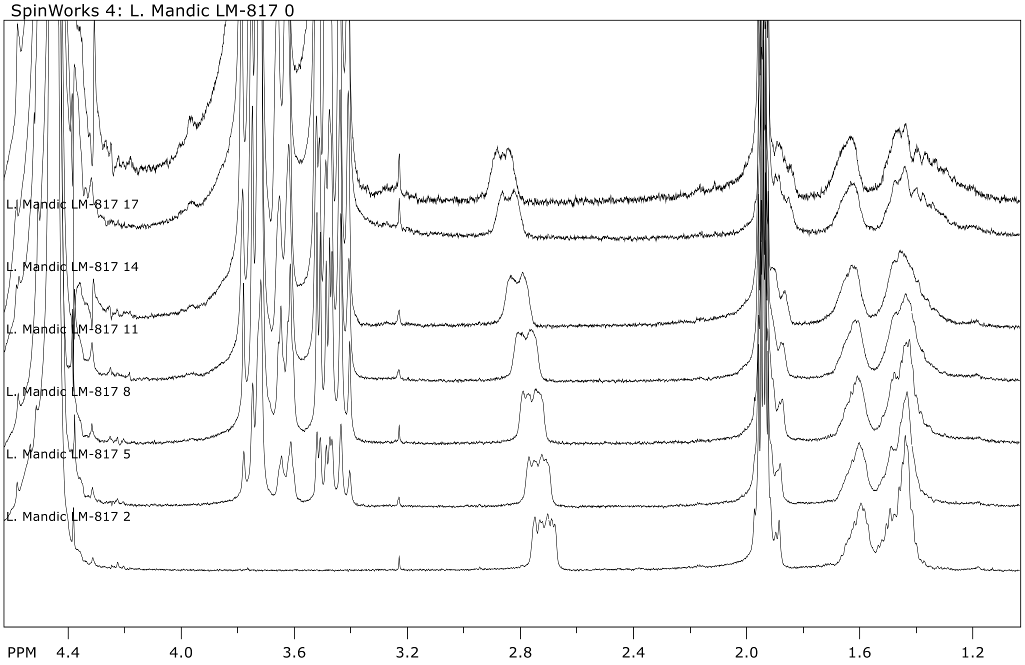

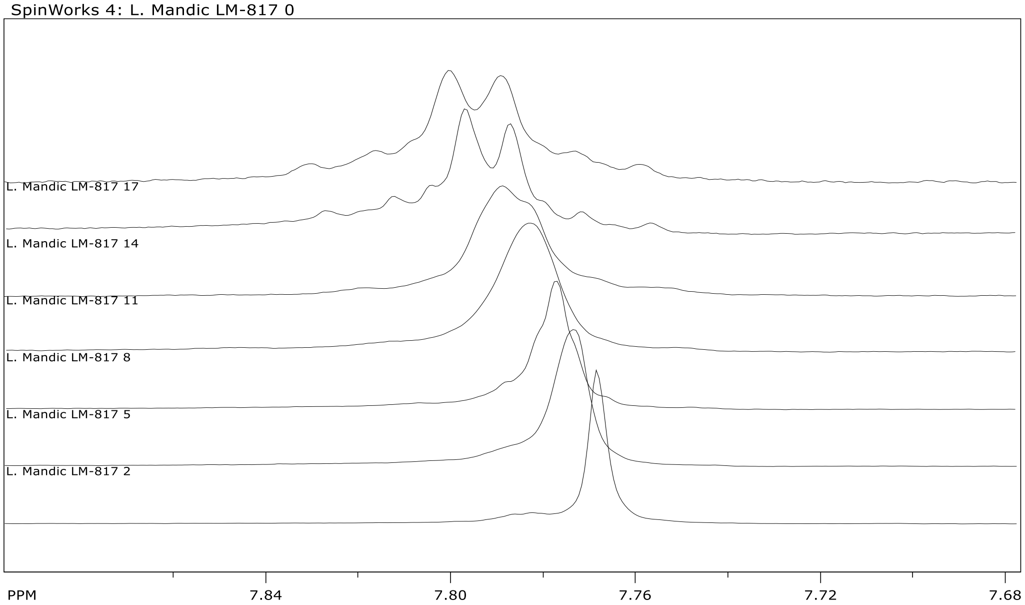

Figure S1. Parts of the ^{1}H NMR spectra of **2** in CD_3CN/D_2O showing signals corresponding to cyclohexane 2 and 6 H-atoms (top) and phthalimide H-atoms (bottom) with increasing β-CD concentration from bottom to top.

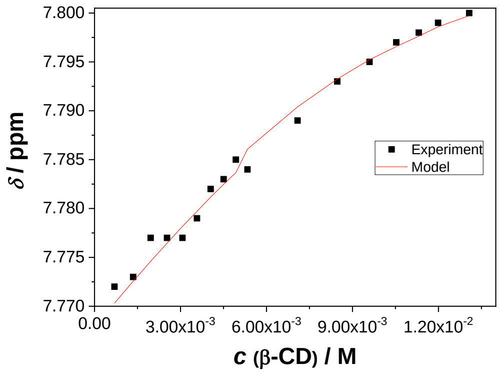

Figure S2. Dependence of the chemical shift of the H-atoms at the phthalimide in compound **2** on β-CD concentration. Dots are experimental values and the red line corresponds to the calculated values by WINEQNMR program [9] according to the model for the formation of 1:1 stoichiometry of the inclusion complex **2@β-CD**.

Table S1. Stability constant for the inclusion complex depending on the H-atom signal which was chosen for nonlinear regression analysis using WinEQNMR software **2**@β-CD.

**Observed H-atom**	K_1 / M^{-1}
Cyclohexane (H at the C-2 and C-6)	190 ± 50
Phthalimide	180 ± 20

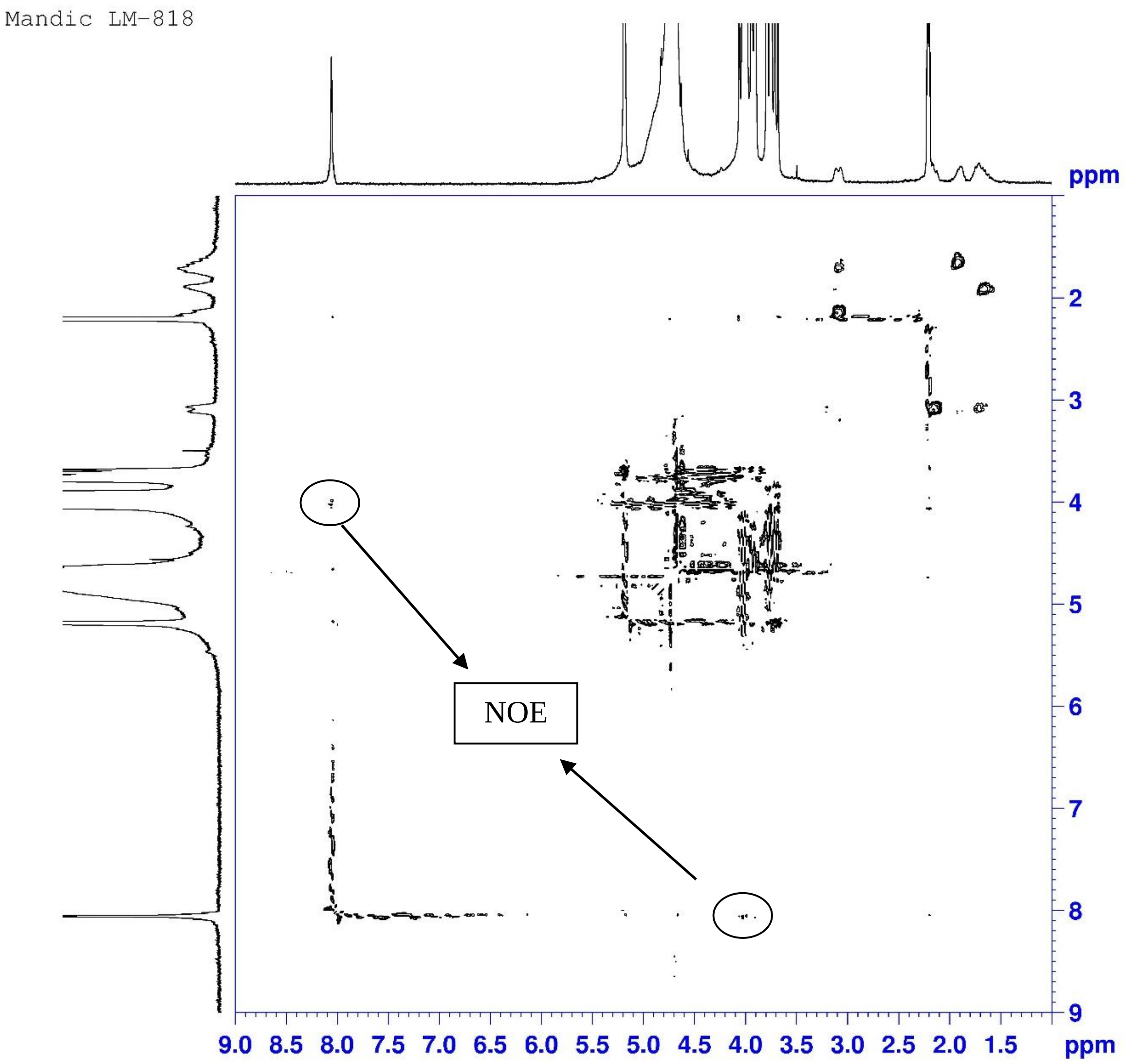

Figure S3. NOESY (CD_3CN-D_2O (3:7), 300 MHz) of **2@β-CD**.

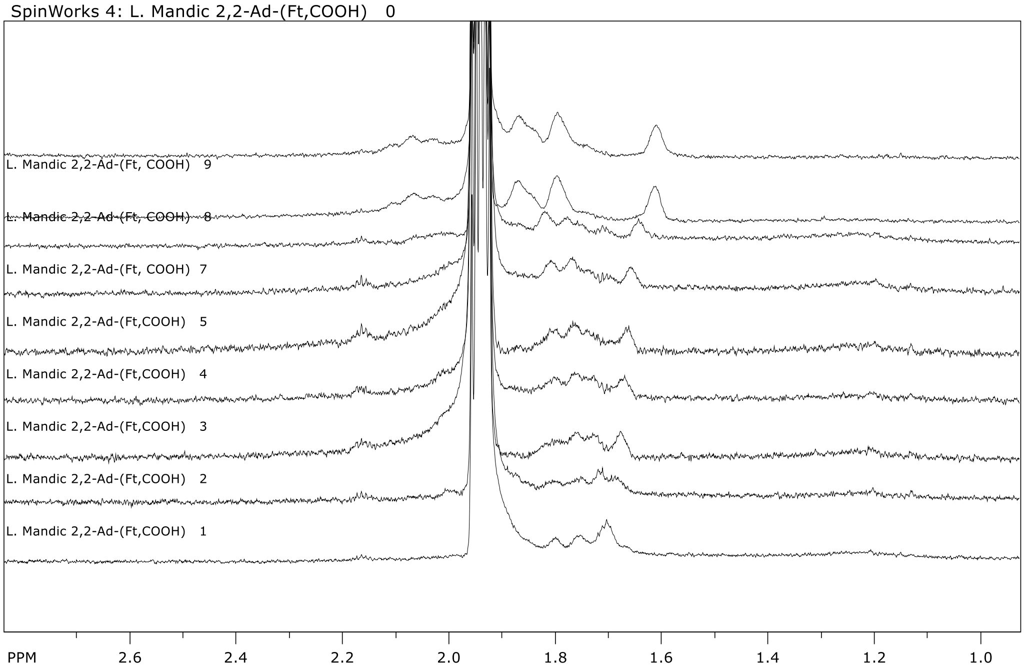

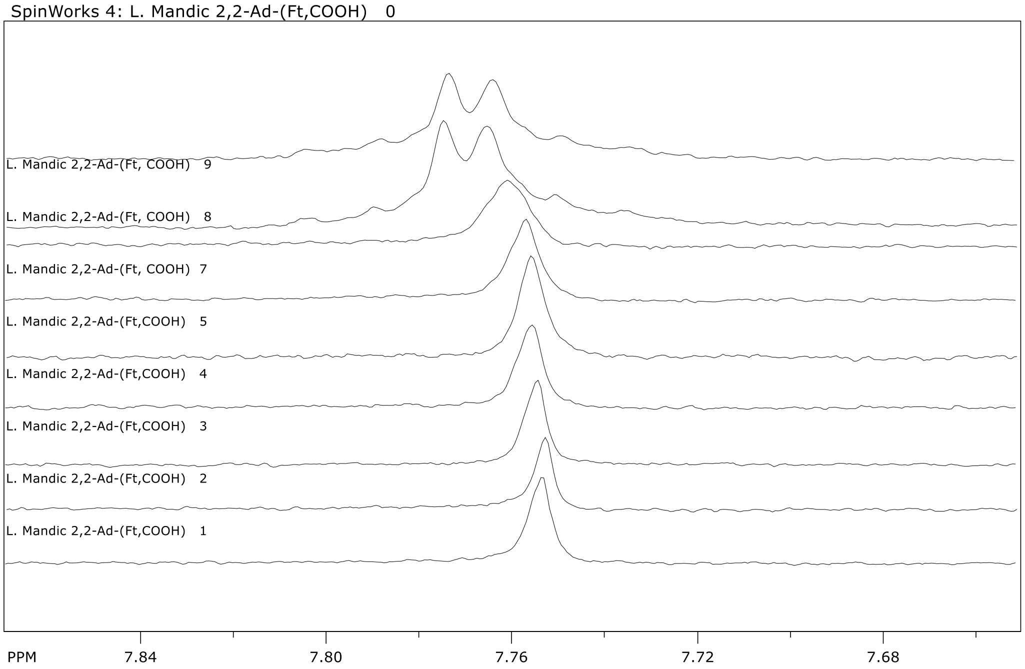

Figure S4. Parts of the ^{1}H NMR spectra of **3** in CD_3CN/D_2O showing signals corresponding to adamantane 6 H-atoms (top) and phthalimide H-atoms (bottom) with increasing β-CD concentration from bottom to top.

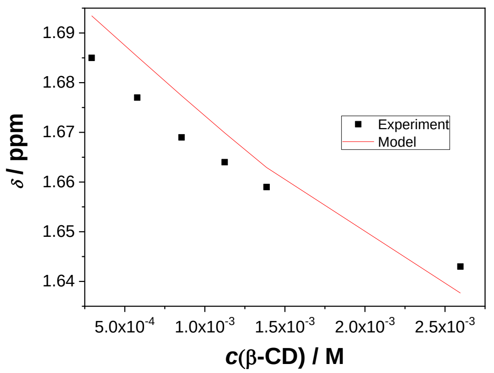

Figure S5. Dependence of the chemical shift of the H-atoms at the phthalimide in compound **3** on β-CD concentration. Dots are experimental values and the red line corresponds to the calculated values by WINEQNMR program according to the model for the formation of 1:1 stoichiometry of the inclusion complex **3@β-CD**.

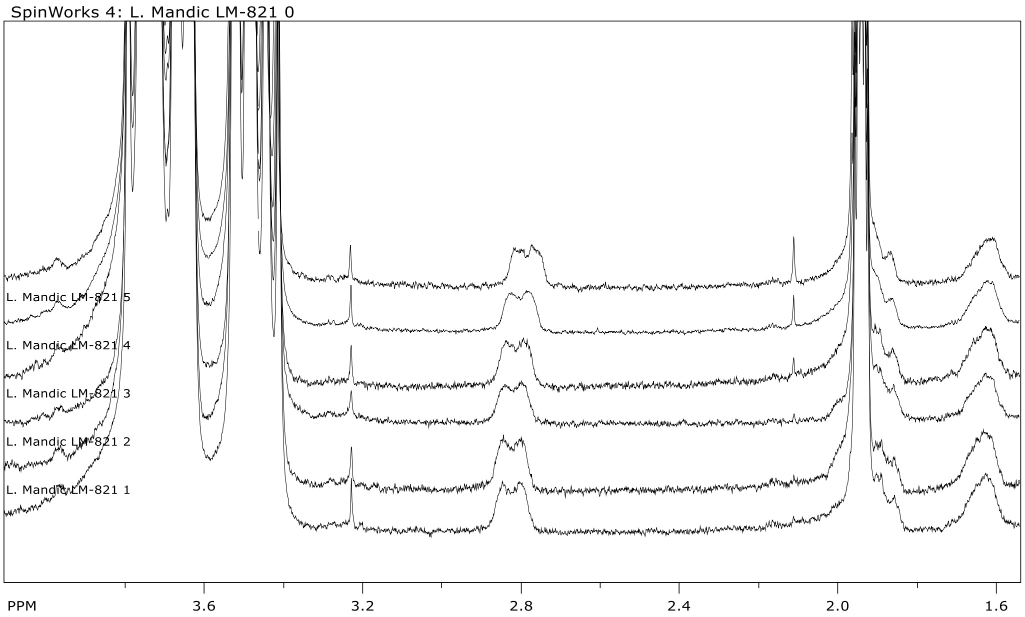

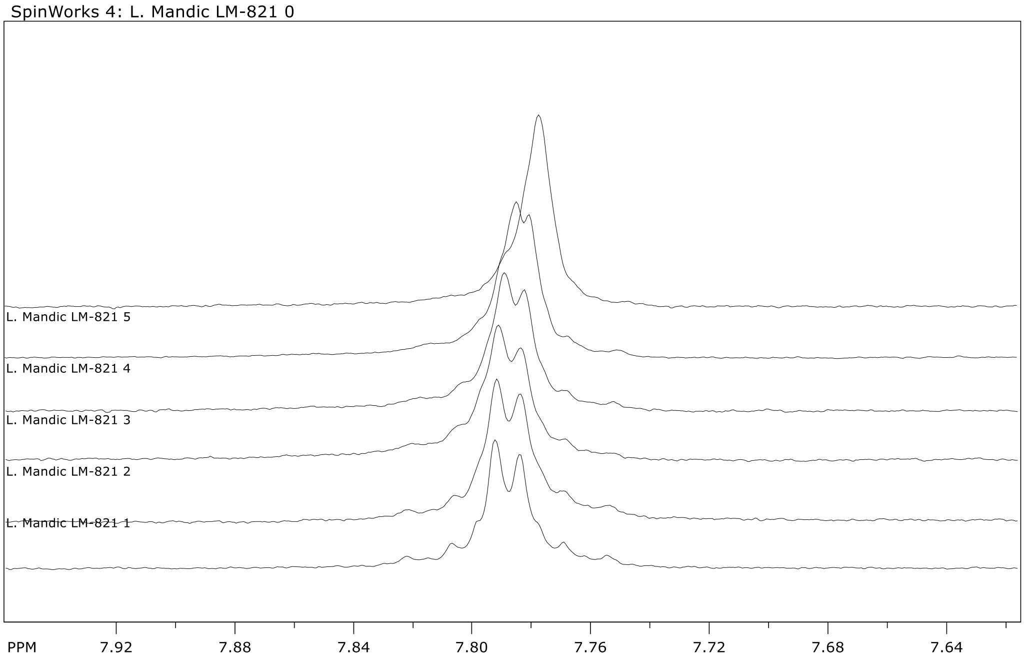

Figure S6. Parts of the ^{1}H NMR spectra of **2@β-CD** in CD_3CN/D_2O showing signals corresponding to the cyclohexane 2 and 6 H-atoms (top) and phthalimide H-atoms (bottom) with increasing acrylonitrile (AN) concentration from bottom to top.

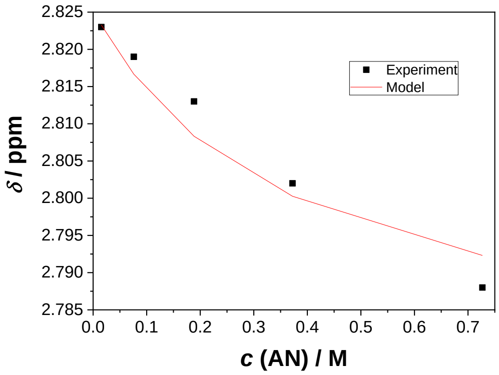

Figure S7. Dependence of the chemical shift of the H-atoms at the cyclohexane 2 and 6-position in the complex **2@β-CD** on acrylonitrile (AN) concentration. Dots are experimental values and the red line corresponds to the calculated values by WINEQNMR program according to the model for the formation of 1:1:1 stoichiometry of the inclusion complex **AN@2@β-CD**.

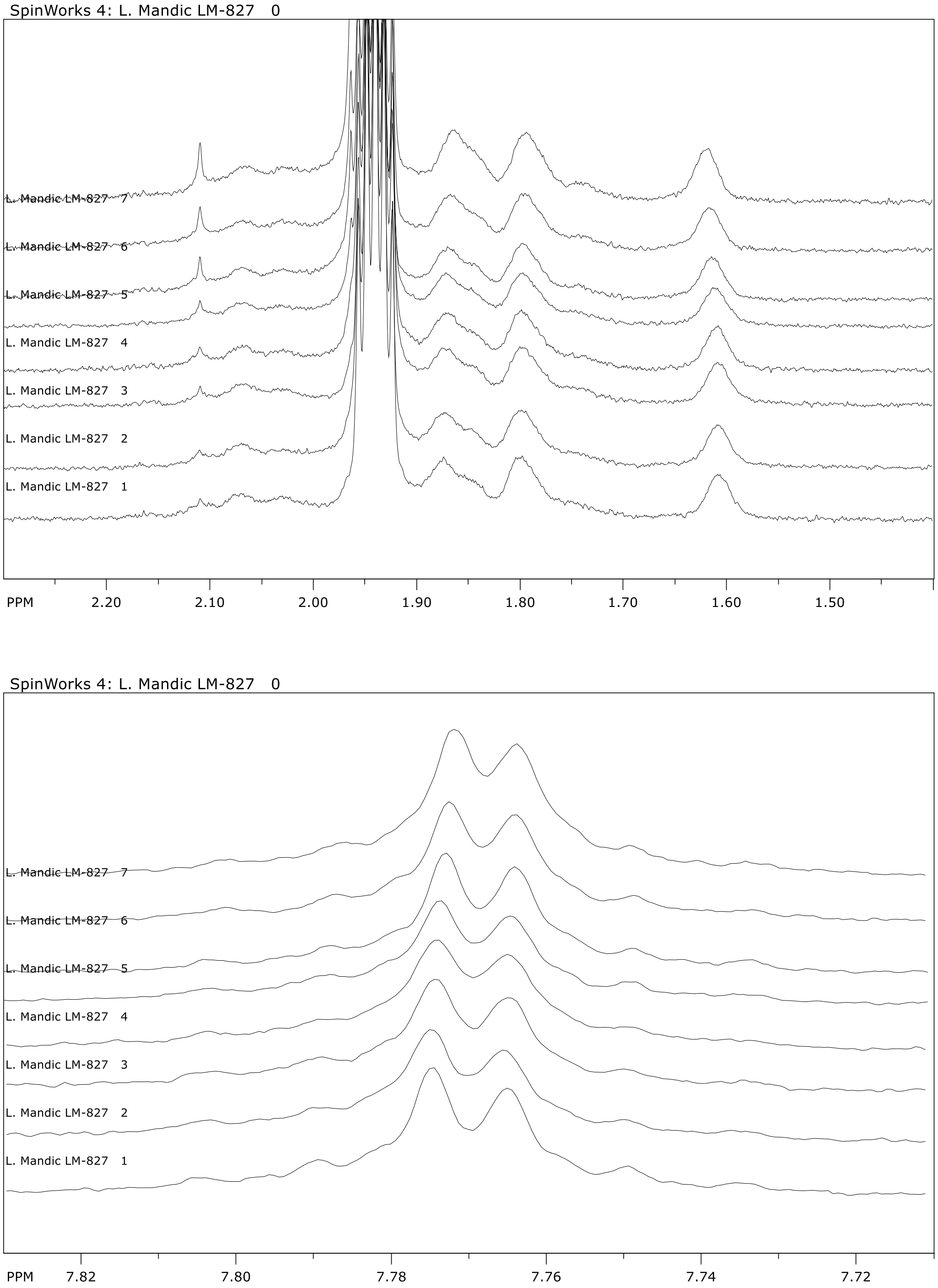

Figure S8. Parts of the ^{1}H NMR spectra of **3@β-CD** in CD_3CN/D_2O showing signals corresponding to the adamantane 6 position (top) and phthalimide H-atoms (bottom) with increasing acrylonitrile concentration from bottom to top.

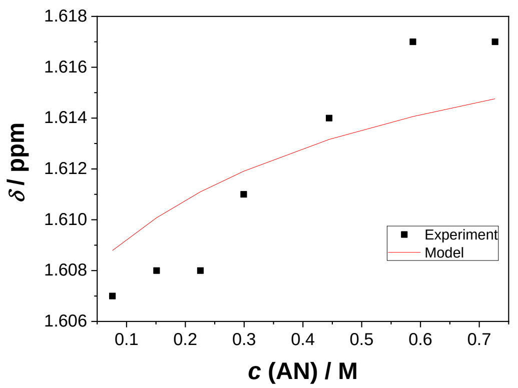

Figure S9. Dependence of the chemical shift of the H-atoms at the adamantane 6-position in the complex **3@β-CD** on AN concentration. Dots are experimental values and the red line corresponds to the calculated values by WINEQNMR program according to the model for the formation of 1:1:1 stoichiometry of the inclusion complex **AN@3@β-CD**.

3. NMR spectra

^{1}H ($CDCl_3$, 600 MHz) and ^{13}C ($CDCl_3$, 150 MHz) NMR spectra of **1**.

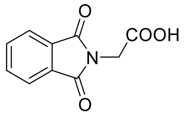

1

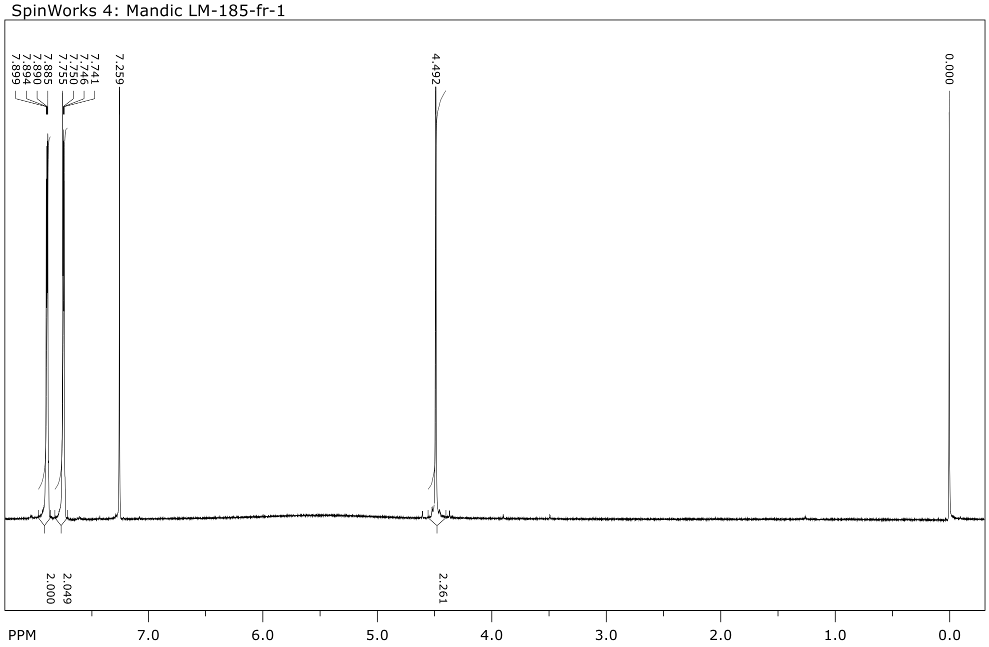

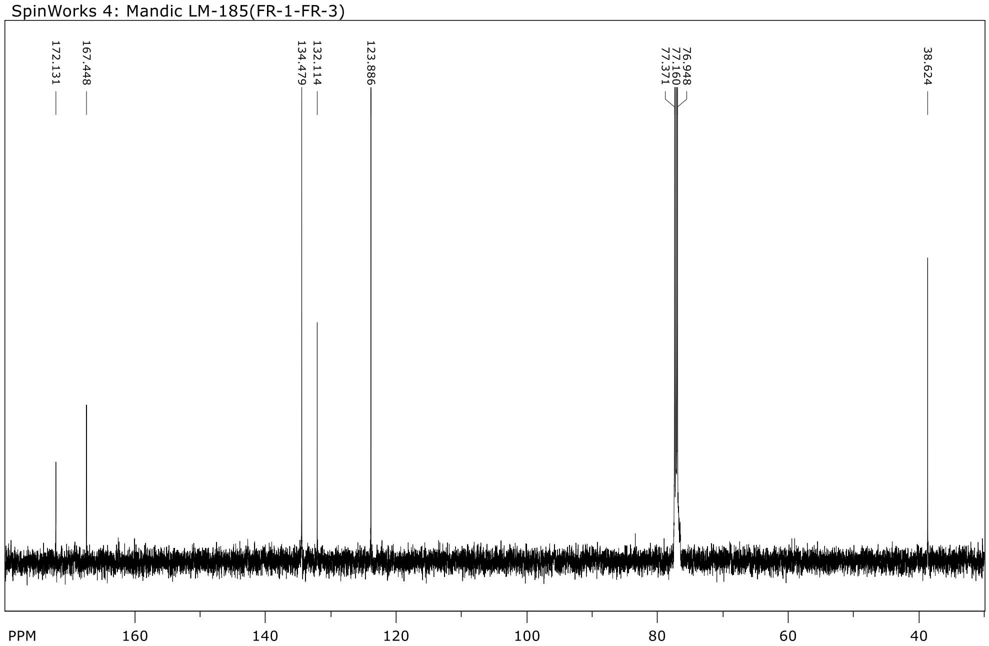

^{1}H ($CDCl_3$, 300 MHz) and ^{13}C ($CDCl_3$, 150 MHz) NMR spectra of **2**.

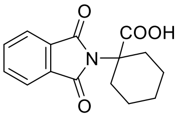

2

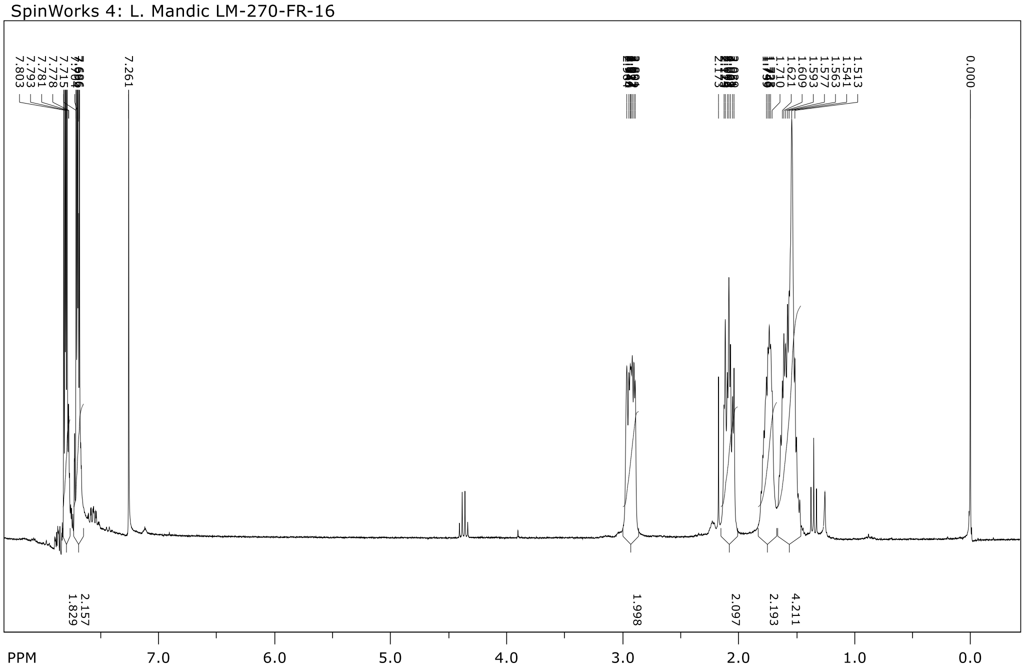

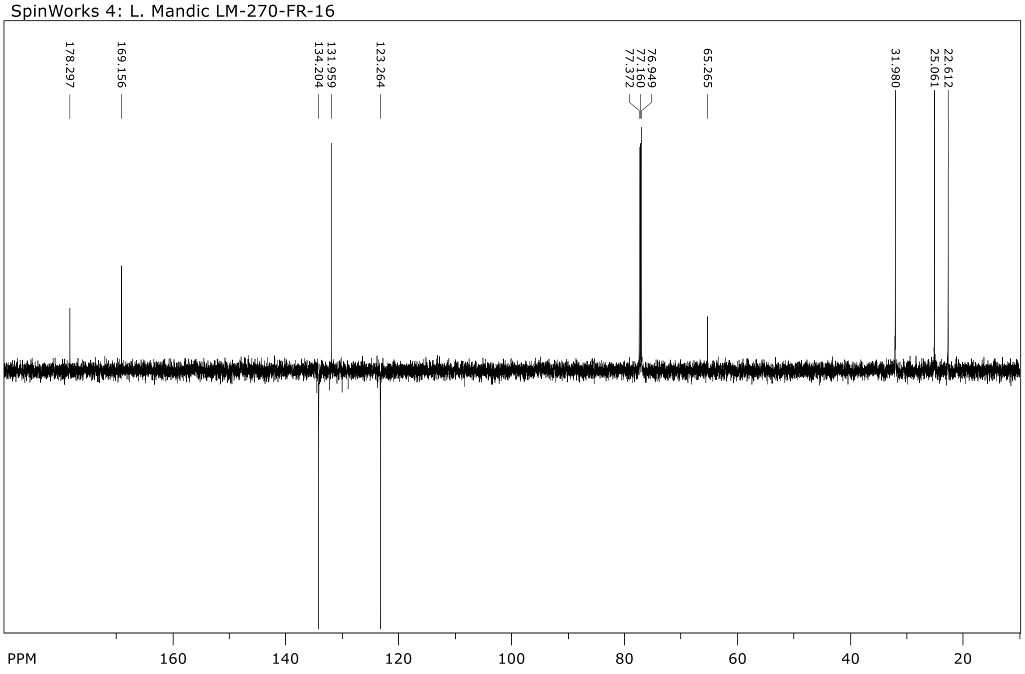

^1H (DMSO-d_6, 300 MHz) and ^{13}C (DMSO-d_6, 75 MHz) NMR spectra of **3**.

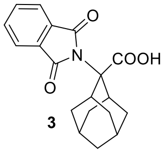

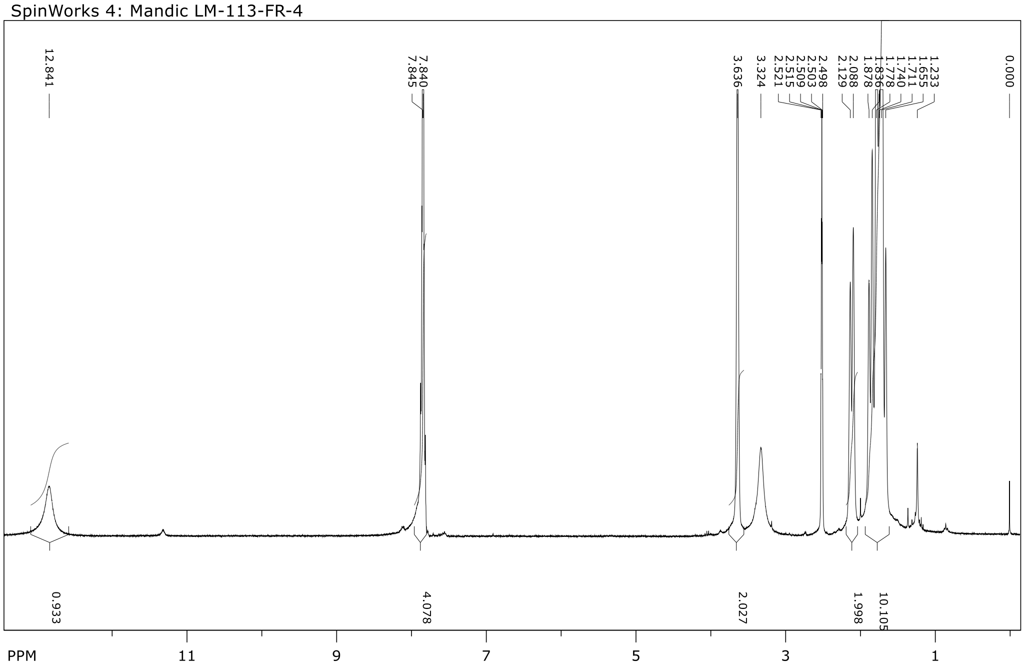

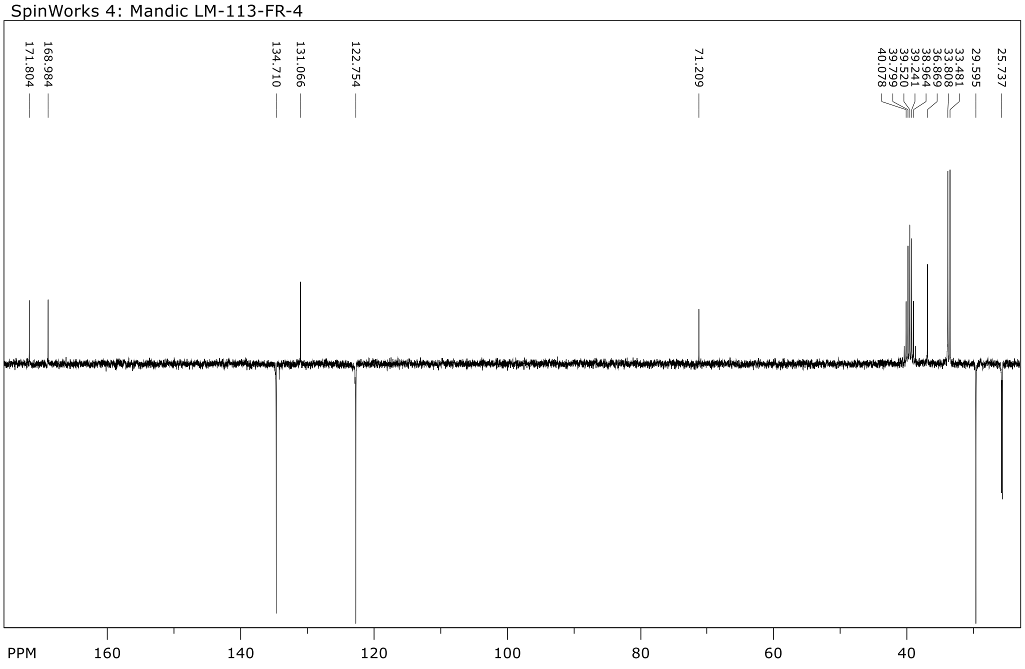

^{1}H ($CDCl_3$, 300 MHz) and ^{13}C ($CDCl_3$, 75 MHz) NMR spectra of **4**.

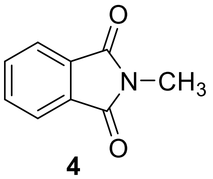

4

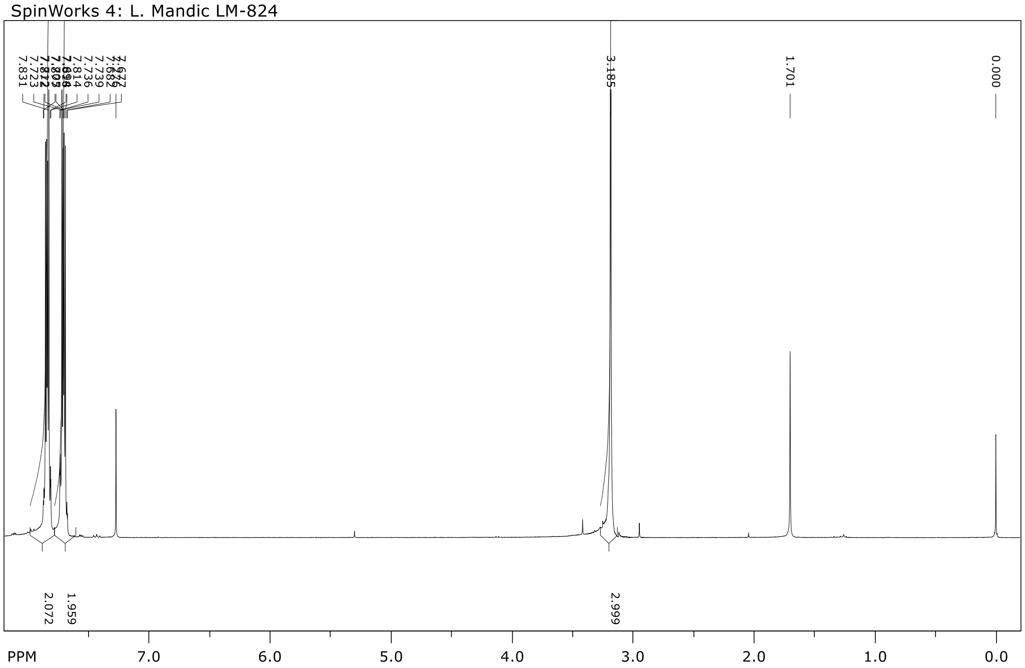

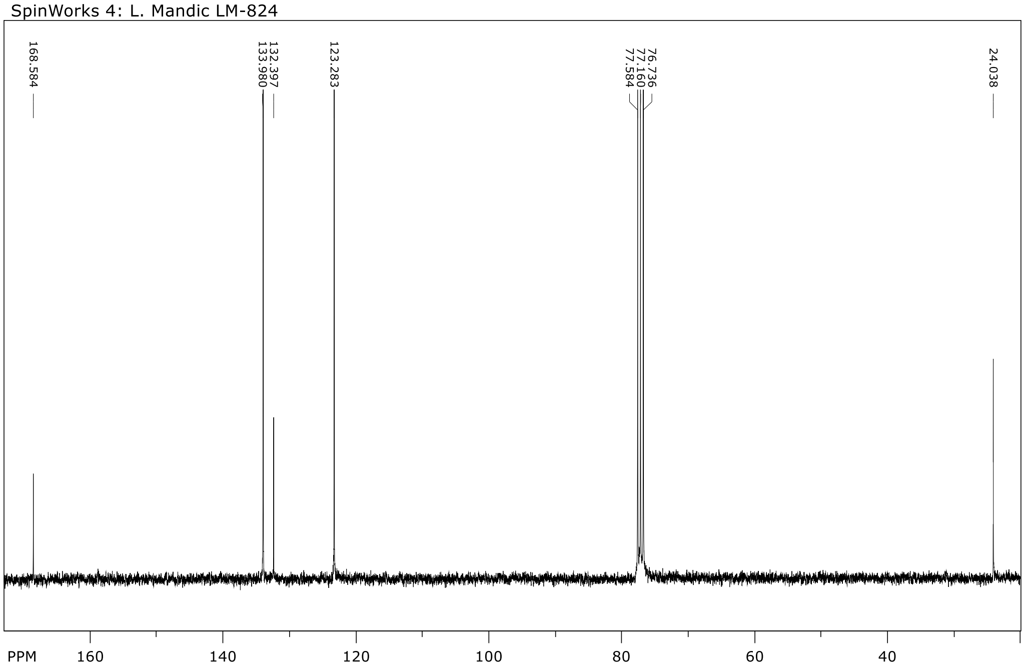

^{1}H ($CDCl_3$, 300 MHz) and ^{13}C ($CDCl_3$, 75 MHz) NMR spectra of **6**.

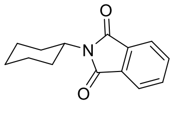

6

SpinWorks 4: L. Mandic LM-cikloheksilftalamid

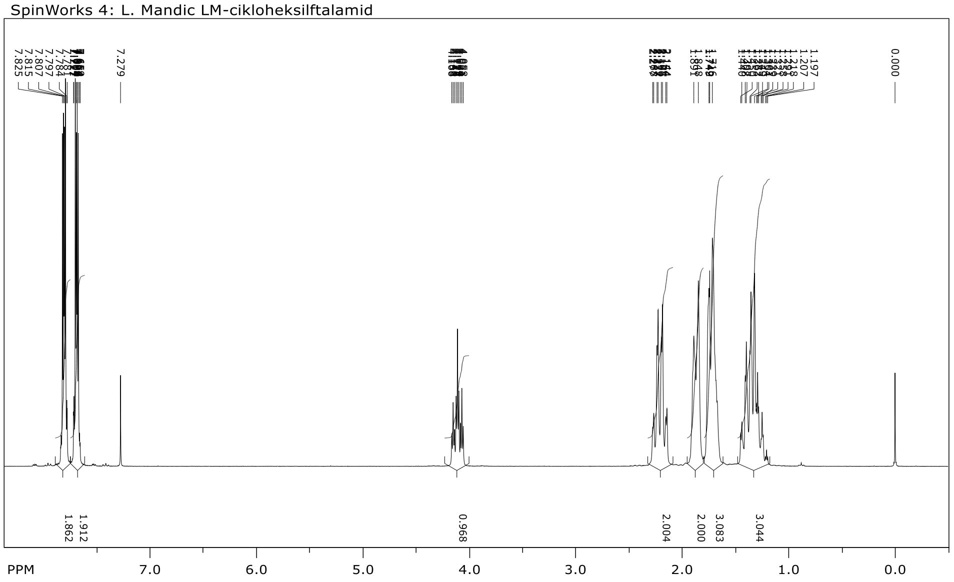

file: K:\LM-315 - K-1 (kloroform)\1\fid expt: <zg30>
transmitter freq.: 300.132701 MHz
time domain size: 32768 points
width: 6172.84 Hz = 20.5670 ppm = 0.188380 Hz/pt
number of scans: 12

freq. of 0 ppm: 300.130000 MHz
processed size: 32768 complex points
LB: 0.300 GF: 0.0000

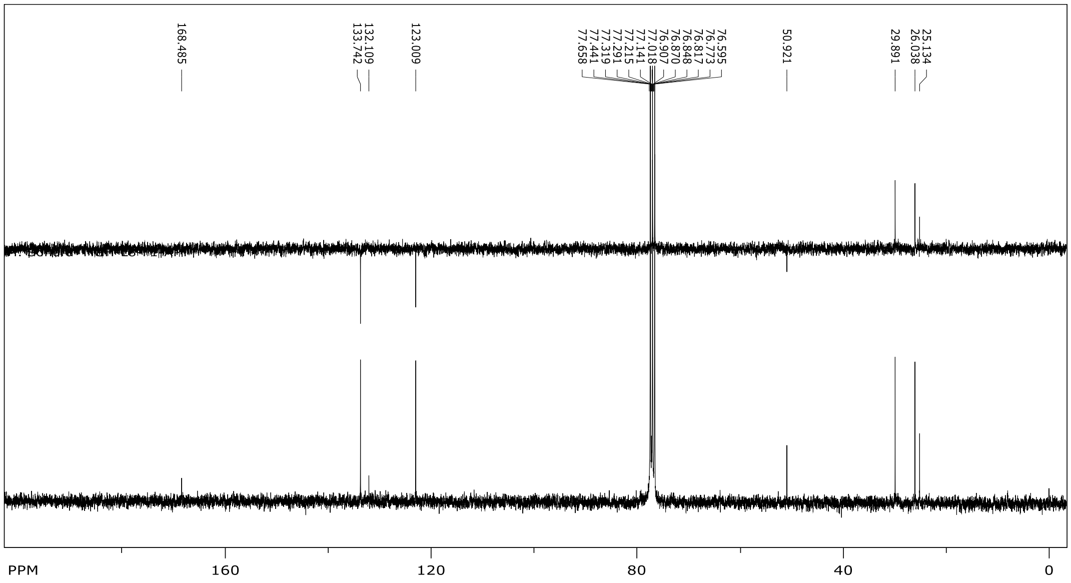

file: ...f1-apt (dekarboksilirani CH)\2\fid expt: <zgpg30>
transmitter freq.: 75.475295 MHz
time domain size: 32768 points
width: 17985.61 Hz = 238.2980 ppm = 0.548877 Hz/pt
number of scans: 3282

freq. of 0 ppm: 75.467747 MHz
processed size: 32768 complex points
LB: 1.000 GF: 0.0000

^{1}H NMR (CD_3OD, 600 MHz) spectrum of **7.**

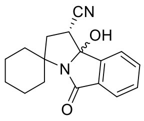

7

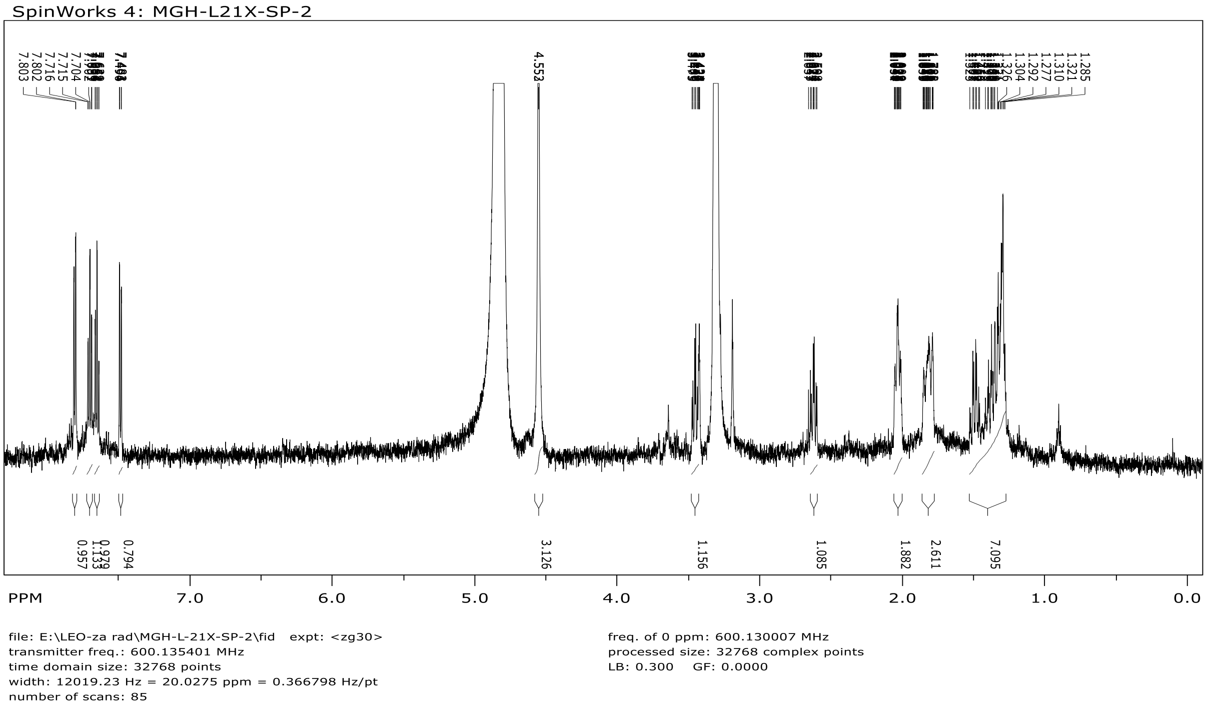

^{1}H NMR (CD_3OD, 300 MHz) and ^{13}C NMR (CD_3OD, 75 MHz) spectrum of **8.**

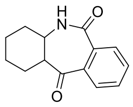

8

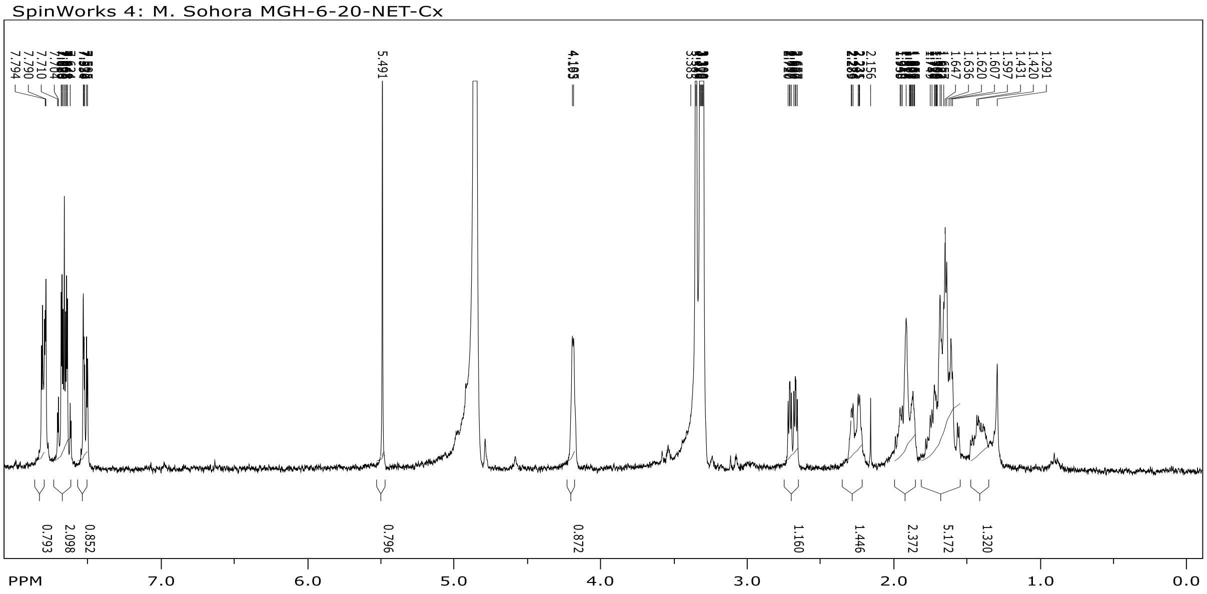

file: ...2018\L-20 čišćenje\L-20-net-Cx\fid expt: <zg30>
transmitter freq.: 300.132701 MHz
time domain size: 32768 points
width: 6172.84 Hz = 20.5670 ppm = 0.188380 Hz/pt
number of scans: 22

freq. of 0 ppm: 300.130005 MHz
processed size: 32768 complex points
LB: 0.500 GF: 0.0000

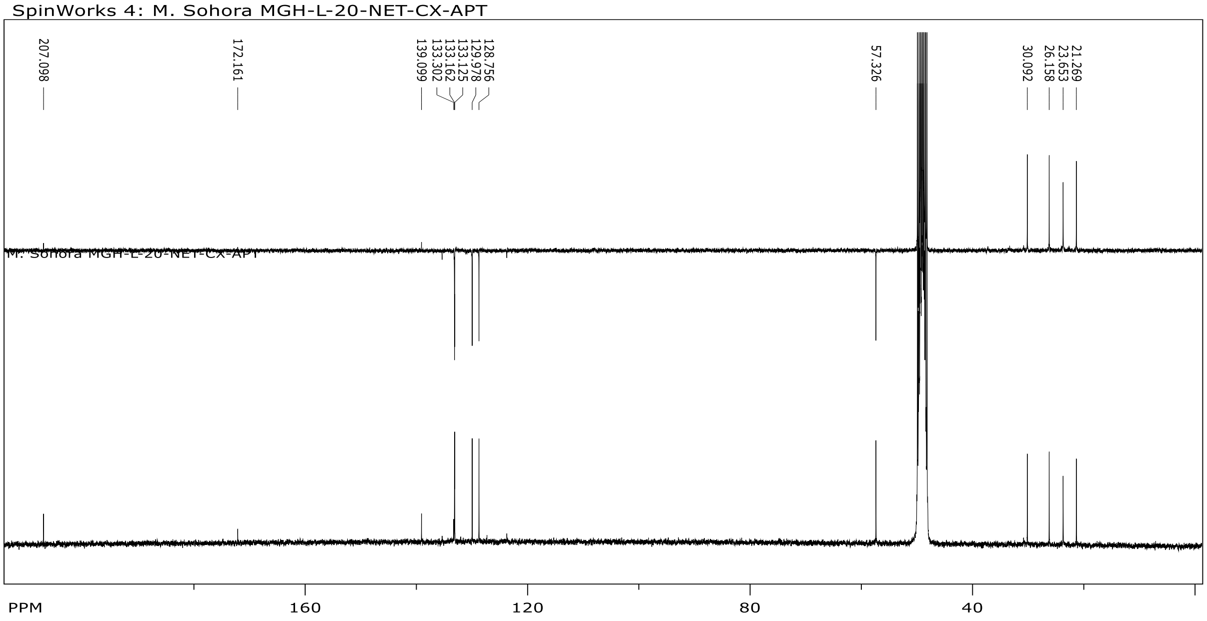

file: ...KLOHEKSANSKI\L-20-net-cx-apt\2\fid expt: <zgpg30>
transmitter freq.: 75.475295 MHz
time domain size: 32768 points
width: 17985.61 Hz = 238.2980 ppm = 0.548877 Hz/pt
number of scans: 213257

freq. of 0 ppm: 75.467641 MHz
processed size: 32768 complex points
LB: 1.000 GF: 0.0000

^{1}H NMR (CD_3OD, 300 MHz) and ^{13}C NMR spectrum (CD_3OD, 75 MHz) spectrum of **9.**

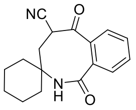

9

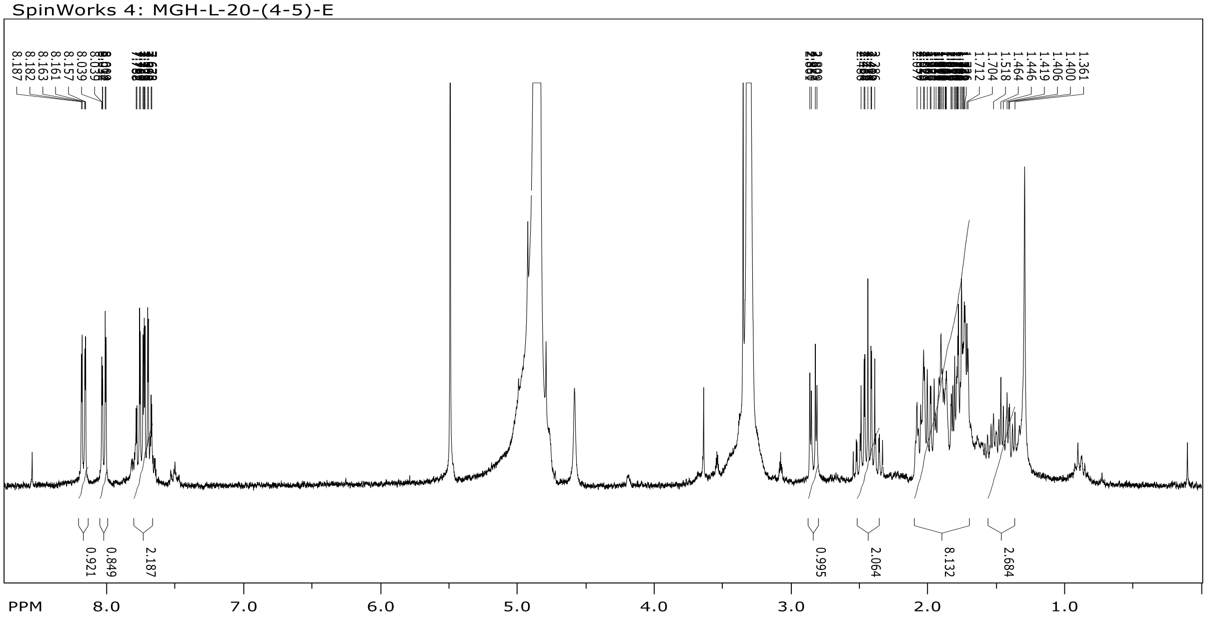

file: ...018\L-20 čišćenje\L-20-(4-5)-E\fid   expt: <zg30>
transmitter freq.: 300.132701 MHz
time domain size: 32768 points
width: 6172.84 Hz = 20.5670 ppm = 0.188380 Hz/pt
number of scans: 128

freq. of 0 ppm: 300.130005 MHz
processed size: 32768 complex points
LB: 0.300   GF: 0.0000

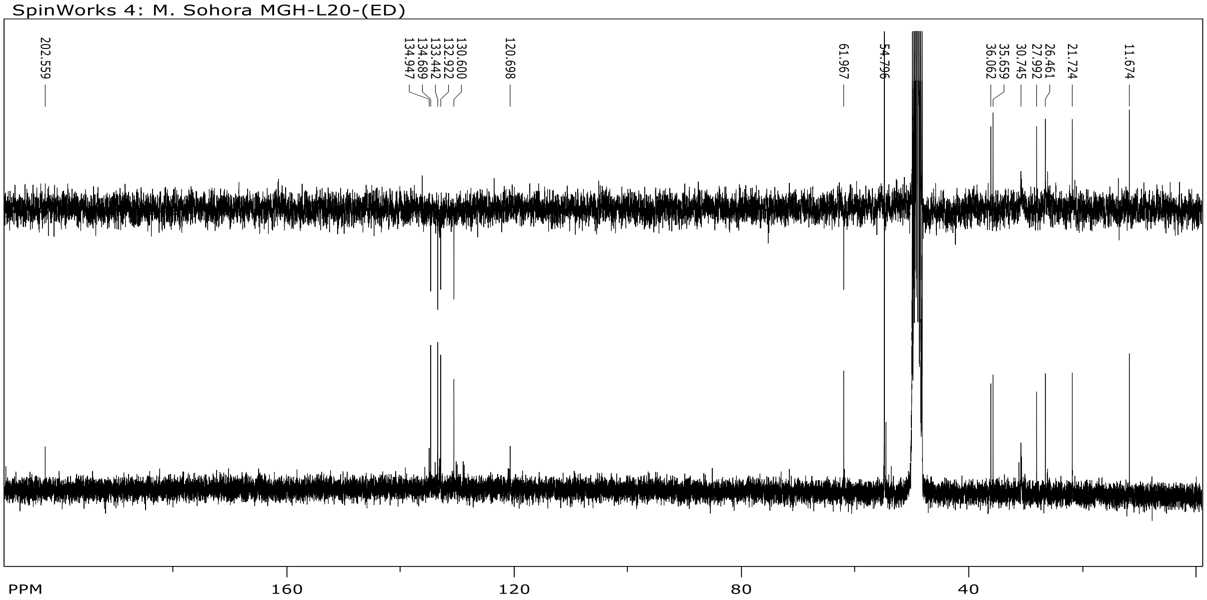

file: ...9\LEO-CIKLOHEKSANSKI\L-20-ed\3\fid   expt: <zgpg30>
transmitter freq.: 75.475295 MHz
time domain size: 32768 points
width: 17985.61 Hz = 238.2980 ppm = 0.548877 Hz/pt
number of scans: 104387

freq. of 0 ppm: 75.467642 MHz
processed size: 32768 complex points
LB: 0.300   GF: 0.0000

^{1}H ($CDCl_3$, 600 MHz) and ^{13}C ($CDCl_3$, 150 MHz) NMR spectra of **10**.

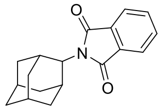

10

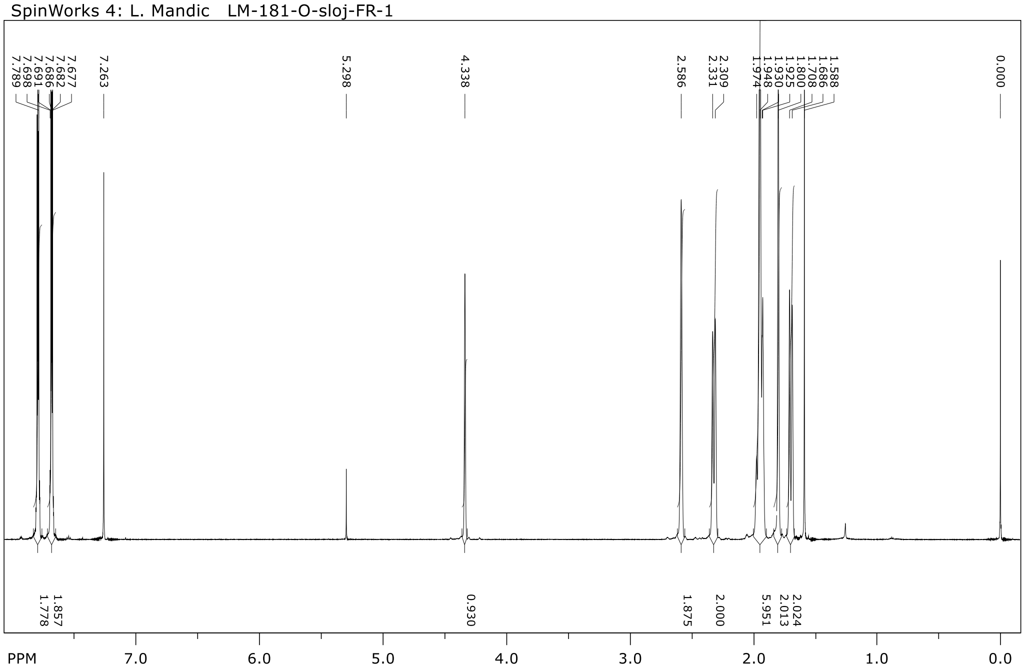

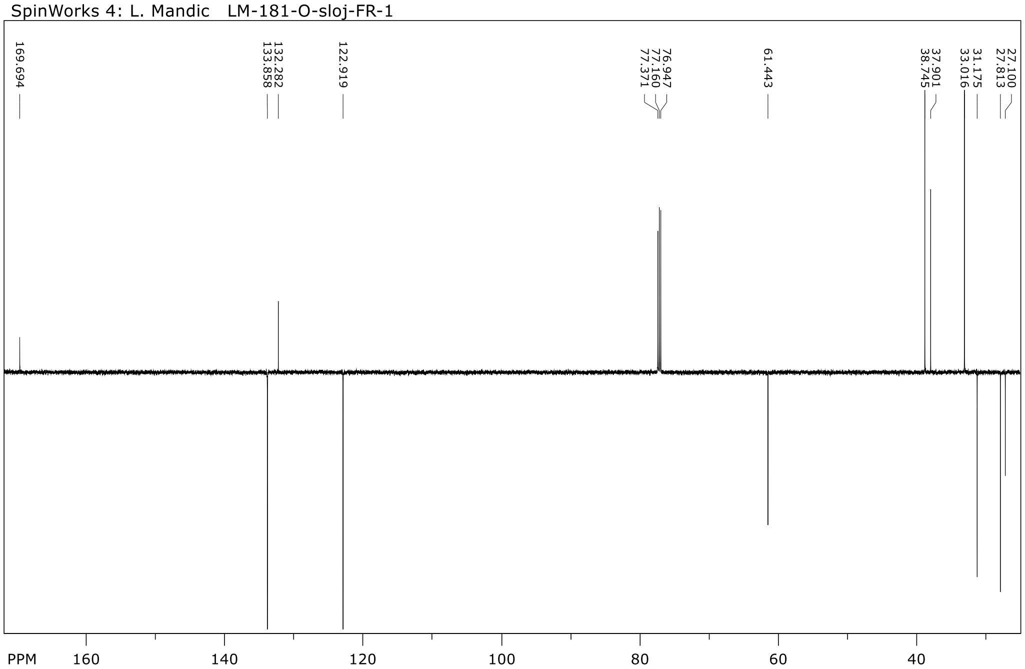

^{1}H NMR (CD_3OD, 600 MHz) and ^{13}C NMR (CD_3OD, 150 MHz) spectrum of **11.**

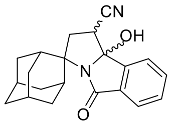

11

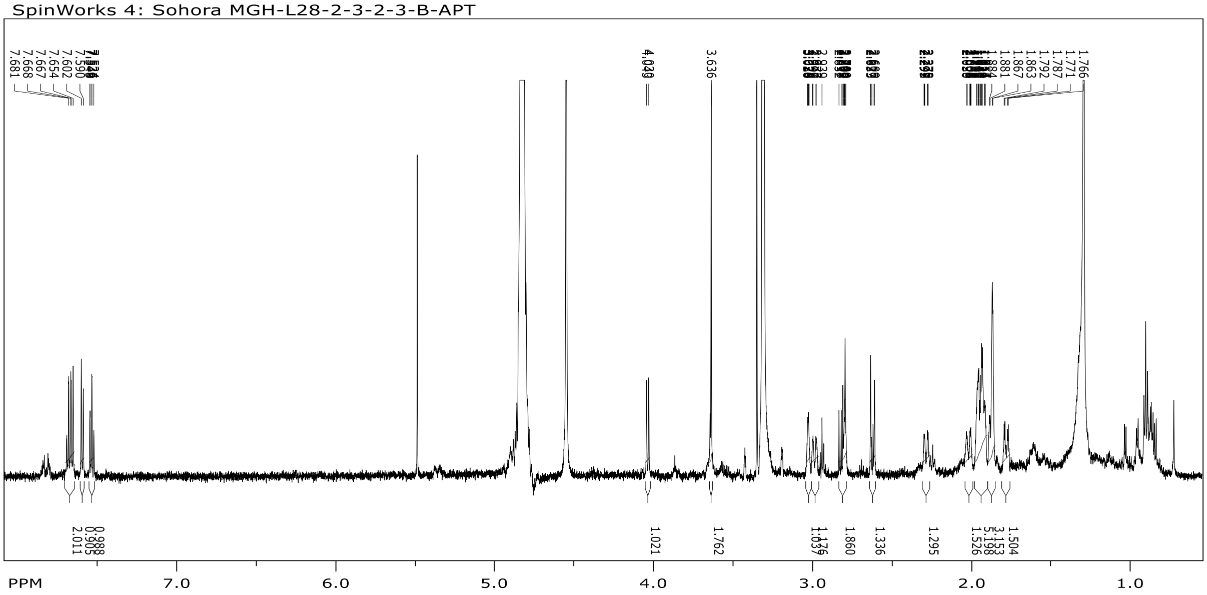

file: ...9\leo\L-28-(2-3)-(2-3)-b-apt\2\fid expt: <zg30>
transmitter freq.: 600.135401 MHz
time domain size: 32768 points
width: 12019.23 Hz = 20.0275 ppm = 0.366798 Hz/pt
number of scans: 32

freq. of 0 ppm: 600.129108 MHz
processed size: 32768 complex points
LB: 0.000 GF: 0.0000

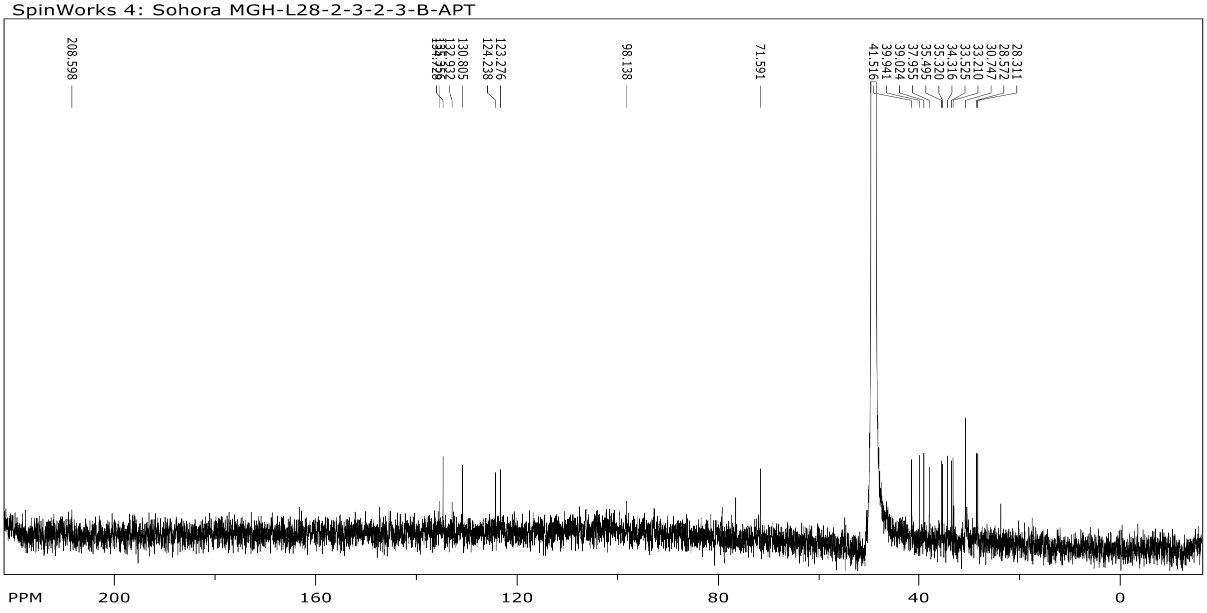

file: ...9\leo\L-28-(2-3)-(2-3)-b-apt\1\fid expt: <zgpg30>
transmitter freq.: 150.917899 MHz
time domain size: 65536 points
width: 35971.22 Hz = 238.3496 ppm = 0.548877 Hz/pt
number of scans: 28738

freq. of 0 ppm: 150.902369 MHz
processed size: 32768 complex points
LB: 3.000 GF: 0.0000

^{1}H NMR ($CDCl_3$, 600 MHz) and ^{13}C NMR ($CDCl_3$, 150 MHz) spectrum of **12.**

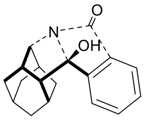

12

SpinWorks 2.5: SOK Margareta MGH-12/3/pk

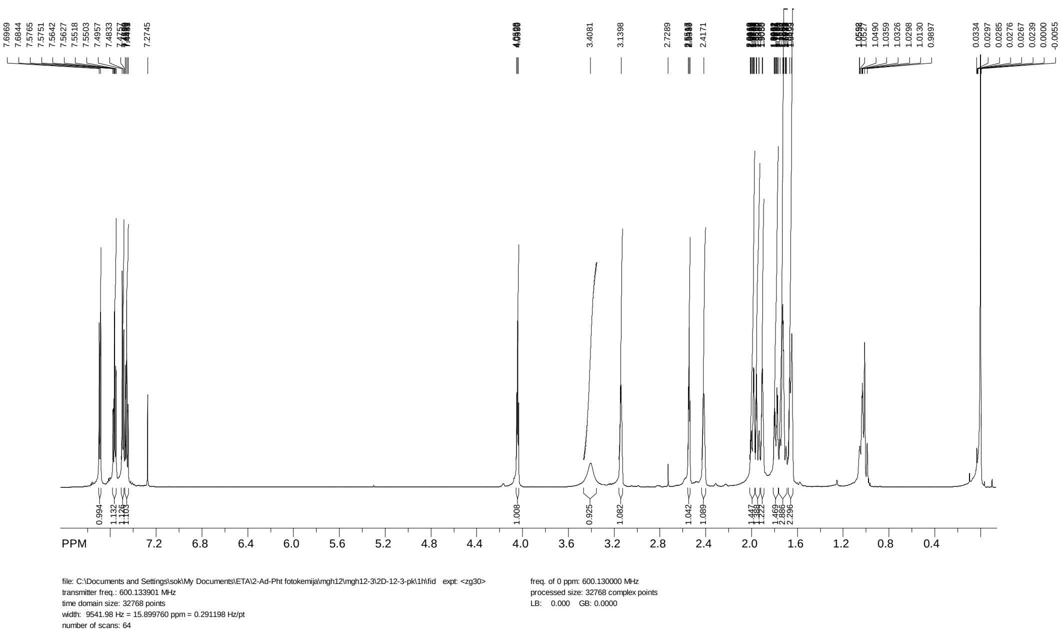

SpinWorks 2.5: SOK Margareta MGH-12/3/pk

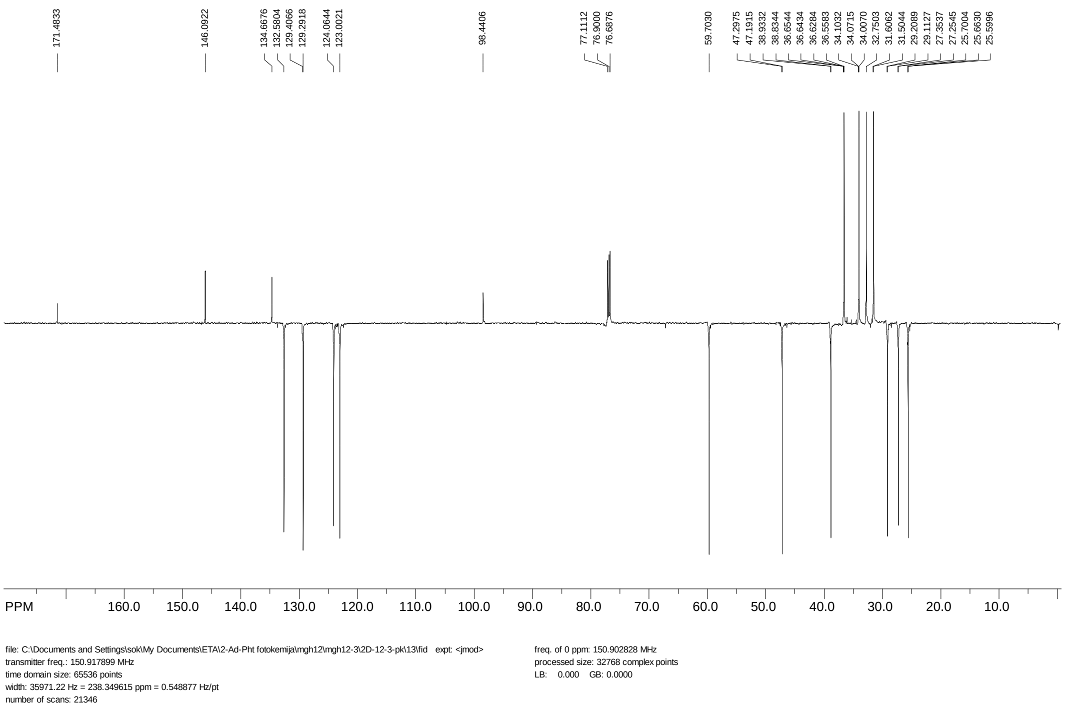

^{1}H NMR ($CDCl_3$, 600 MHz) and ^{13}C NMR ($CDCl_3$, 150 MHz) spectrum of **13.**

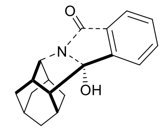

13

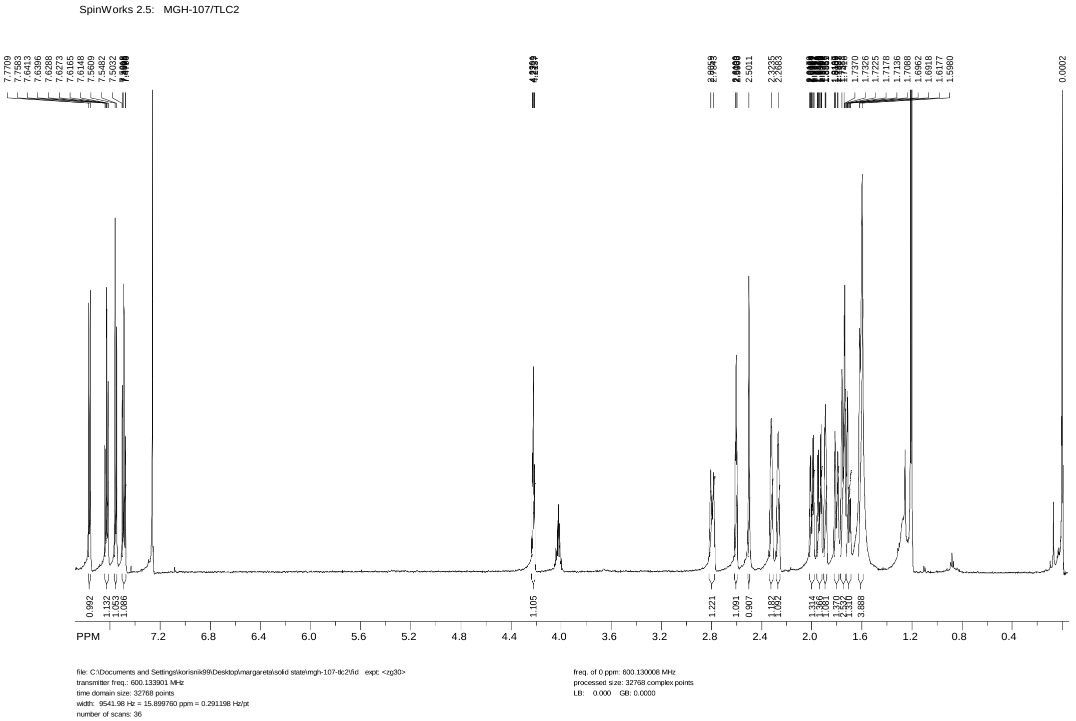

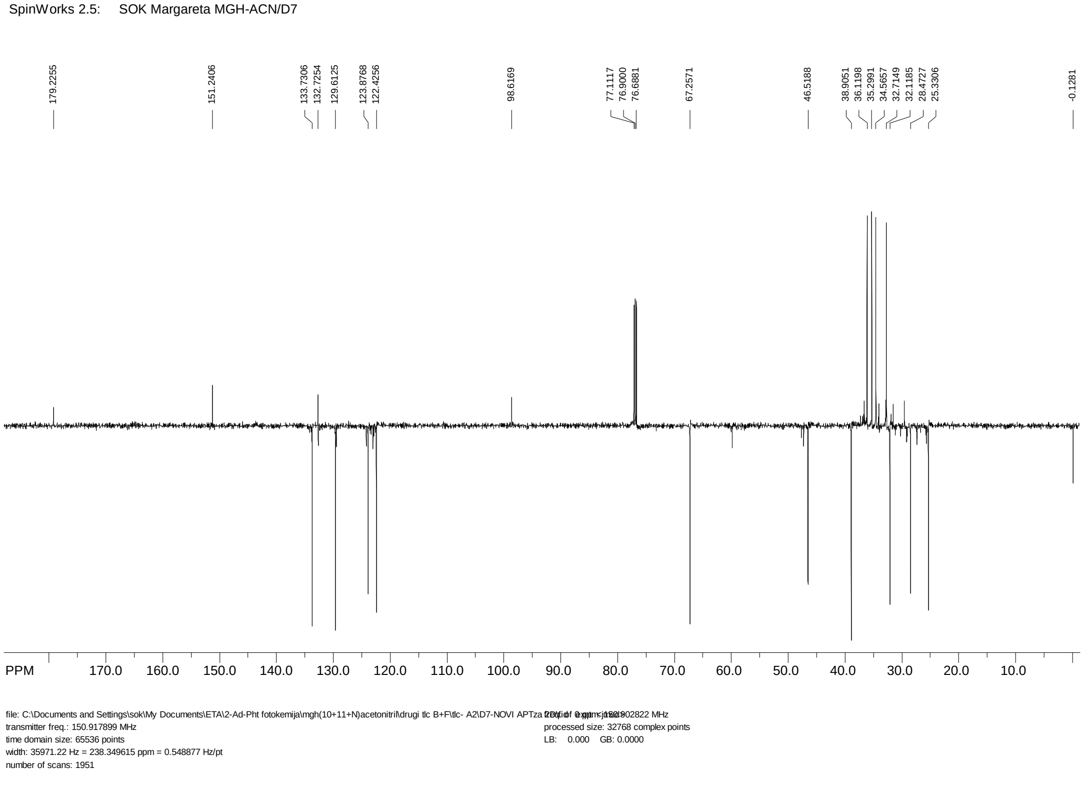

^{1}H NMR ($CDCl_3$, 600 MHz) and ^{13}C NMR ($CDCl_3$, 150 MHz) spectrum of **14.**

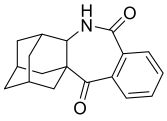

14

SpinWorks 2.5: SOK Horvat MGH-12/2/D

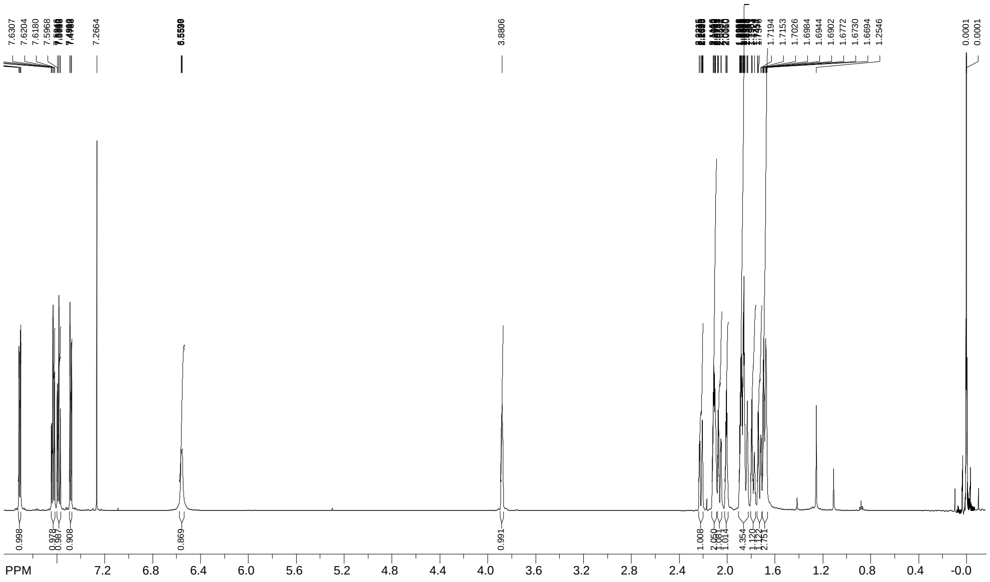

file: C:\Documents and Settings\sok\My Documents\ETA\2-Ad-Pht fotokemija\mgh12\mgh12-2\TLC\12-2-D -1H ZA 2D SPEKTAR\fid expt: <zg30> freq. of 0 ppm: 600.130005 MHz
transmitter freq.: 600.133901 MHz processed size: 32768 complex points
time domain size: 32768 points LB: 0.000 GB: 0.0000
width: 9541.98 Hz = 15.899760 ppm = 0.291198 Hz/pt
number of scans: 64

SpinWorks 2.5: SOK M.Horvat MGH-12/2/D

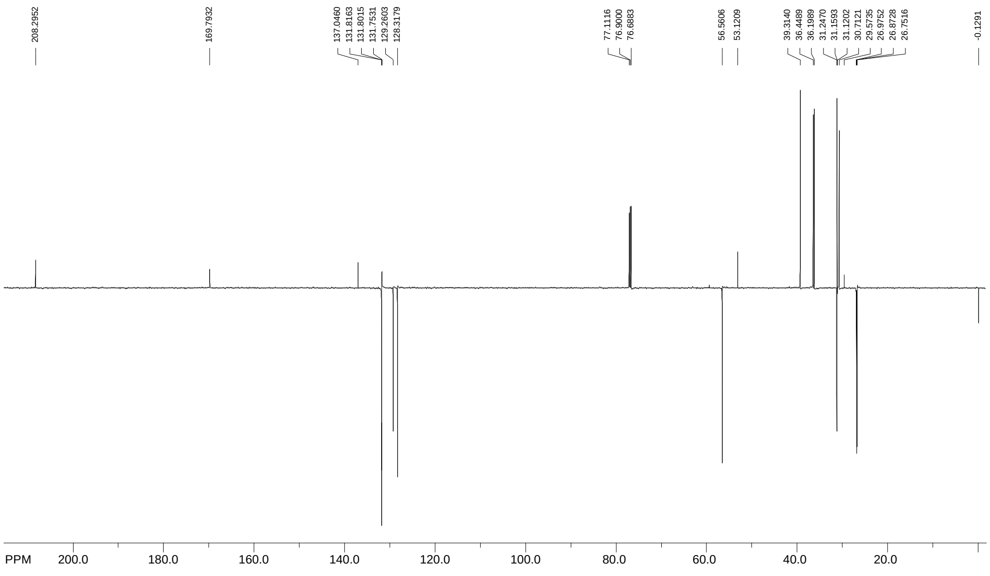

file: C:\Documents and Settings\sok\My Documents\ETA\2-Ad-Pht fotokemija\mgh12\mgh12-2\TLC\12-2-D-13C ZA 2D SPEKTAR\fid expt: [illegible] freq. of 0 ppm: 150.902824 MHz
transmitter freq.: 150.917899 MHz processed size: 32768 complex points
time domain size: 65536 points LB: 0.000 GB: 0.0000
width: 35971.22 Hz = 238.349615 ppm = 0.548877 Hz/pt
number of scans: 6739

4. References

[1] Vamecq, J.; van Derpoorten, K.; Poupaert, J. H.; Balzarini, J.; De Clercq E.; Stables, J. P., *Life Sci.*, **1998**, *63*, PL267-PL274.

[2] Racine, S.; Hegedüs, B.; Scopelliti R.; Waser, J., *Chem. Eur. J.*, **2016**, *22*, 11997-12001.

[3] Mandić, L.; Mlinarić-Majerski, K.; Griesbeck, A. G.; Basarić, N., *Eur. J. Org. Chem.*, **2016**, 4404-4414.

[4] Horvat, Š.; Mlinarić-Majerski, K.; Glavaš-Obrovac, Lj.; Jakas, A.; Veljković, J.; Marczi, S.; Kragol, G.; Roščić, M.; Matković, M.; Milostić-Srb, A., *J. Med. Chem.*, **2006**, *49*, 3136-3142.

[5] Wang, L.; Fu, H.; Jiang, Y.; Zhao, Y., *Chem. Eur. J.*, **2008**, *14*, 10722-10726.

[6] Yoon, U. C.; Kim, D. U.; Lee, C. W.; Choi, Y. S.; Lee, Y.-J.; Ammon, H. L.; Mariano, P. S., *J. Am. Chem. Soc.*, **1995**, *117*, 10, 2698-2710.

[7] Worlikar, S. A.; Larock, R. C., *J. Org. Chem.*, **2008**, *73*, 7175-7180.

[8] Basarić, N.; Horvat, M.; Mlinarić-Majerski, K.; Franković, O.; Neudörfl, J.; Griesbeck, A. G., *Tetrahedron*, **2009**, *65*, 1438-1443.

[9] Hynes, M. J., *J. Chem. Soc. Dalton Trans.*, **1993**, 311-312.
